# Associative pyridinium electrolytes for air-tolerant redox flow batteries

**DOI:** 10.1038/s41586-023-06664-7

**Published:** 2023-11-29

**Authors:** Mark E. Carrington, Kamil Sokołowski, Erlendur Jónsson, Evan Wenbo Zhao, Anton M. Graf, Israel Temprano, Jade A. McCune, Clare P. Grey, Oren A. Scherman

**Affiliations:** 1 Yusuf Hamied Department of Chemistry, University of Cambridge, Cambridge, UK; 2 Melville Laboratory for Polymer Synthesis, Yusuf Hamied Department of Chemistry, University of Cambridge, Cambridge, UK; 3 Present Address: Magnetic Resonance Research Center, Institute for Molecules and Materials, Faculty of Science, Radboud University Nijmegen, Nijmegen, The Netherlands

**Keywords:** Energy, Electrochemistry

## Abstract

Pyridinium electrolytes are promising candidates for flow-battery-based energy storage^1–4^. However, the mechanisms underlying both their charge–discharge processes and overall cycling stability remain poorly understood. Here we probe the redox behaviour of pyridinium electrolytes under representative flow battery conditions, offering insights into air tolerance of batteries containing these electrolytes while providing a universal physico-chemical descriptor of their reversibility. Leveraging a synthetic library of extended bispyridinium compounds, we track their performance over a wide range of potentials and identify the singlet–triplet free energy gap as a descriptor that successfully predicts the onset of previously unidentified capacity fade mechanisms. Using coupled operando nuclear magnetic resonance and electron paramagnetic resonance spectroscopies^5,6^, we explain the redox behaviour of these electrolytes and determine the presence of two distinct regimes (narrow and wide energy gaps) of electrochemical performance. In both regimes, we tie capacity fade to the formation of free radical species, and further show that *π*-dimerization plays a decisive role in suppressing reactivity between these radicals and trace impurities such as dissolved oxygen. Our findings stand in direct contrast to prevailing views surrounding the role of *π*-dimers in redox flow batteries^1,4,7–11^ and enable us to efficiently mitigate capacity fade from oxygen even on prolonged (days) exposure to air. These insights pave the way to new electrolyte systems, in which reactivity of reduced species is controlled by their propensity for intra- and intermolecular pairing of free radicals, enabling operation in air.

## Abstract (web summary)

The redox behaviour of pyridinium electrolytes under representative flow battery conditions is investigated, offering insights into air tolerance of batteries containing these electrolytes while providing a universal physico-chemical descriptor of their reversibility.

## Main

Among redox flow battery (RFB) active materials, those based on organic molecules are expected to provide both substantial cost benefits over existing, largely vanadium-based chemistries and more pathways forward for improvements in energy density through chemical diversity^12–15^. Showing high aqueous solubility, negative potentials and good electrochemical stability under neutral conditions, viologens (that is, 4,4′ bispyridinium species)^4^ have been demonstrated as RFB anolytes in a variety of single- and double-electron couples^8,16–19^. Despite these promising characteristics, a systematic understanding of their electrochemical and degradative processes under representative flow battery conditions is lacking. With most bispyridinium-based RFBs to date having been run under strict air-free conditions, little is known about their air tolerance^8,16^. Specifically, the reactivity of reduced bispyridinium species, mono- and diradicals, in RFB systems is critical for battery performance as these species are known to readily transfer electrons to dissolved oxygen^4^. This can lead to the formation of reactive oxygen species detrimental to battery components and the electrolytes themselves. Moreover, formation of radical-mediated assembled viologen structures such as *π*-dimers, *σ*-dimers and charge-transfer complexes (Supplementary Fig. 1)^20^ have been previously linked to capacity fade^1,4,7–11^, favouring subsequent molecular design intended to avoid such structures. In the present report, we explore structural features of a diverse library of bispyridinium electrolytes alongside operando metrologies to demonstrate insights into air tolerance of batteries containing these electrolytes. These insights provide a set of general principles that can be used for the continued improvement of RFB electrolytes.

## Building a library of bispyridinium electrolytes

**Fig. 1:**
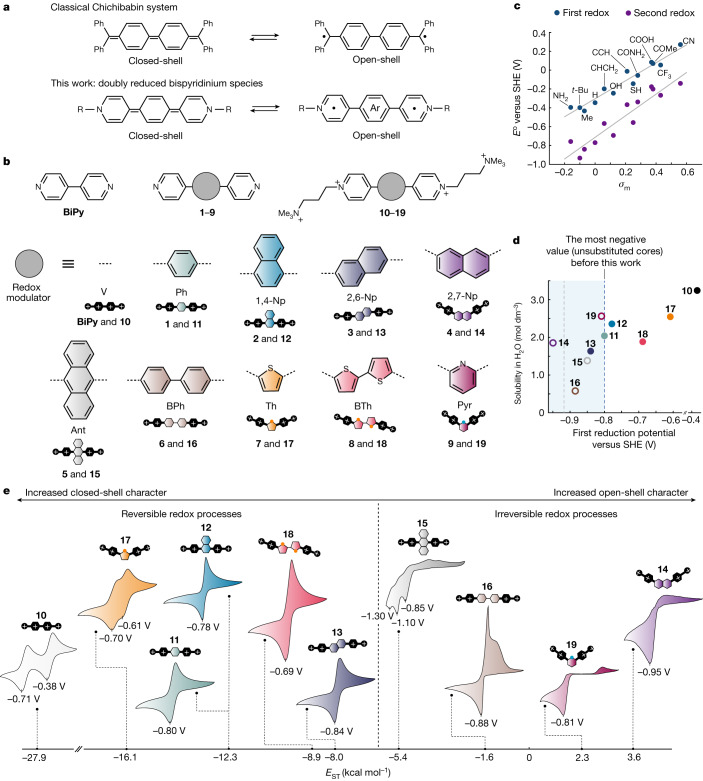
Synthetic library of bispyridinium electrolytes. **a**, Comparison between classical diradical hydrocarbons and bispyridinium diradicals. **b**, Schematic representation of bispyridine cores with a variety of redox modulators (**1**–**9**) and their fully alkylated analogues, the subject of this work (**10**–**19**). **BiPy** refers to classical 4,4′-bipyridine. **c**, Linear correlations obtained between DFT-calculated redox potential values and tabulated experimental *meta* Hammett constant values (*σ*_m_)^27^ for R-groups introduced to pyridinium nitrogens. Trendlines indicate least-squares linear fits obtained for first and second redox events. **d**, Plot of solubility versus first reduction potential for compounds **10**–**19**. Filled circles indicate electrochemically reversible compounds. Empty circles indicate electrochemically irreversible compounds. The dashed line (grey) indicates the lowest reduction potential reached by a bispyridinium RFB electrolyte: substituted or unsubstituted. The shaded region indicates compounds with reduction potentials below those of any bispyridinium electrolyte featuring an unsubstituted core reported so far. **e**, Voltammograms for compounds **10**–**19** ordered by their respective singlet–triplet gap (*E*_ST_) values. Potentials are referenced to the SHE. Previously reported compounds **10**, **11** and **17** were included as positive controls for classical viologen as well as extended homocyclic and heterocyclic bispyridiniums, respectively^8,22,23^. For clarity, chloride counterions are omitted in all cases.

The electronic structure of charged bispyridinium compounds can be readily modulated by incorporation of a central aromatic core between pyridinium rings^16,21–23^. We therefore designed and synthesized a library of molecules from which redox characteristics of viologens and ‘extended’ bispyridinums could be systematically investigated (Fig. 1a). Starting from 4-pyridinyl-boronic acid and the corresponding aryl dibromide, Suzuki–Miyaura coupling was used as an efficient diversifying chemistry^24,25^ to prepare extended bipyridines **1**–**9** using both homogeneous and heterogeneous catalytic strategies (Fig. 1b and Supplementary Information Sections 2 and 3). These were then N-alkylated to form 3-trimethylammonium-propyl (TMAP)-functionalized bispyridinium compounds **10**–**19**. As the dependence of viologen redox behaviour on the electron withdrawing or donating character of *N*-alkyl groups is known^26^, elaboration of R-group effects was carried out using a series of density functional theory (DFT)-based computational screens. In all cases, excellent linear correlations were found between tabulated experimental R-group Hammett constants (*σ*_m_, Fig. 1c and Supplementary Table 1)^27^ and calculated redox potentials, with gaps between first and second redox potentials being modulated primarily by core, not R-group chemistry (Supplementary Figs. 5–7). Thus, a synthetic library based on varied core motifs was deemed sufficient for subsequent investigation and the R-group chemistry was limited to TMAP. The final library of compounds consists of molecules with greater and lesser degrees of conjugation, conjugation along different physico-chemical axes, varying aromatic substitution patterns and heteroatom substituted aromatic cores.

Bispyridinium compounds **10**–**19** showed excellent aqueous solubility (up to 3.3 M) and a broad range of electrochemical characteristics (Fig. 1d,e). By cyclic voltammetry, **10**, **11**, **12**, **13**, **17** and **18** showed reversible first potentials spanning −0.35 to −0.82 V versus the standard hydrogen electrode (SHE). Notably, **12** and **13** possessed first redox potentials of −0.77 and −0.82 V, respectively, the most negative for pyridinium RFB electrolytes featuring unsubstituted cores so far. By contrast, **14**, **15**, **16** and **19** were found to show electrochemical processes that were irreversible. As **12**, **13** and **14** are regioisomers of one another, these results indicate that substitution patterns profoundly influence the electronic structure of reduced bispyridinium species. Notably, within this group of viologen derivatives, doubly reduced structures that can adopt a Kekulé structure (**12,** **13**) are reversible, whereas **14**, which cannot, is irreversible. The degree of conjugation was also found to have consequences for reversibility. Compounds showing greater degrees of conjugation generally showed lower reversibility relative to those showing lesser degrees of conjugation (**15** versus **12**, **11**; and **16** versus **11**, respectively). For heterocyclic bispyridiniums **17** and **18**, the loss in reversibility with increased conjugation was much less pronounced relative to that of homocyclic bispyridiniums **11** and **16**. Instead, **17** and **18** were found to be electrochemically more similar to **11** and **13**, suggesting that an ordering principle based on electronic factors rather than conjugation length may be more appropriate. One such principle is the singlet–triplet free energy gap (*E*_ST_).

## Singlet–triplet gap as a physico-chemical descriptor

Bispyridinium compounds generally show closed-shell singlet structures when doubly reduced^4^. However, when conjugation is increased to levels similar to that of **16** (that is, two or more conjugated core rings), a population of thermally accessible triplet diradical states has been proposed (Fig. 1a), the onset of which is typically described using the difference in Gibbs free energy of the corresponding singlet and triplet states, *E*_ST_ (refs. ^28–31^). Notably, incorporating an anthracene extension within bispyridinium molecules (that is, **15**) provides more energy levels allowing formation of triplet excited states even for non-reduced counterparts^25^. Given the irreversibility of compounds featuring non-Kekulé structures when doubly reduced (**14**, **19**), as well as the known diradical character of a closely analogous hydrocarbon (for example, Chichibabin’s hydrocarbon, Fig. 1a) and pyridinium-based compounds^28–31^, a series of DFT calculations was carried out to determine the extent to which the current set of molecules show triplet diradical character, and the consequences, if any, such character has for electrochemical performance.

Calculated *E*_ST_ values for compounds **10**–**19** ranged from −27.9 to +3.6 kcal mol^−1^ (Fig. 1e), with more negative values being indicative of a greater propensity to form closed-shell (singlet) structures. For compounds possessing an *E*_ST_ value less than −6.0 kcal mol^−1^ (**10**, **11**, **12**, **13**, **17**, **18**), voltammetry indicated reversible redox processes with homocyclic-core electrolytes exhibiting  more negative potentials than those with heterocyclic cores. Furthermore, compounds with a greater degree of conjugation showed more negative potentials over analogues with a lower degree of conjugation. Conversely, a loss of reversibility was observed for  compounds with *E*_ST_ values between −6.0 and 0 kcal mol^−1^ (**15**, **16**). Likely, the loss of reversibility arises from the generation of thermally accessible and highly reactive diradical species, and their subsequent participation in parasitic side reactions (for example, proton abstraction, *σ*-dimerization or cyclization^28^) to yield electrochemically irreversible products. At *E*_ST_ > 0 kcal mol^−1^ (**14**, **19**), compounds adopt non-Kekulé doubly reduced structures, and were also found to be irreversible. The *E*_ST_ values thus correlate well with electrochemical irreversibility. In light of these results, the singlet–triplet gap emerges as a useful and versatile physico-chemical descriptor for both comparing bispyridinium compounds and monitoring the onset of electrochemical irreversibility.

## Electrochemical performance and capacity fade

**Fig. 2:**
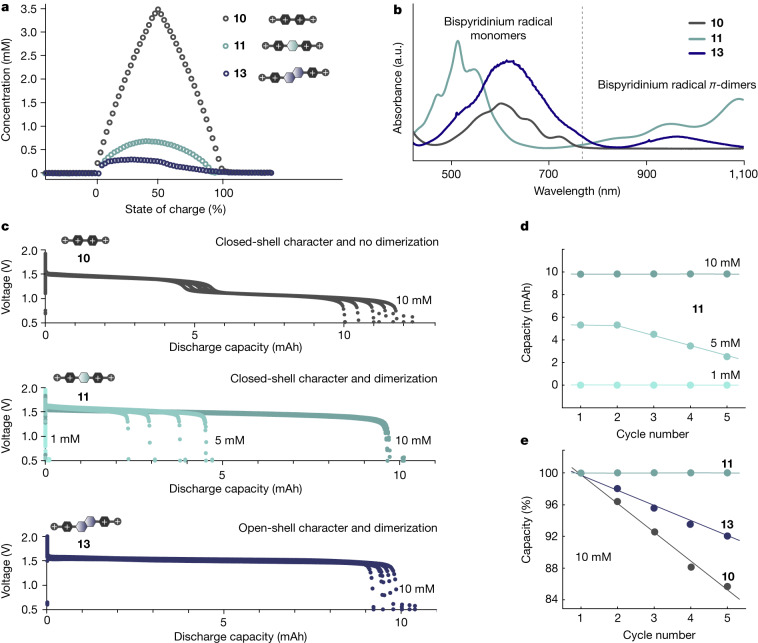
Reduced bispyridinium compounds, their performance characteristics and dimerization propensity. **a**, Radical concentration profiles for **10**, **11** and **13** during charge derived from spin counting based on the EPR data shown in Extended Data Fig. 1. **b**, Spectroelectrochemical data for **10**, **11** and **13** at a concentration of 1 mM. Bands assigned to bispyridinium singly reduced and *π*-dimeric species^40,41^ are shown. **c**, Voltage versus discharge capacity over five full charge–discharge cycles for 10 mM **10**, **11** and **13** in 100 mM NaCl and 20 mM 4-hydroxy-TEMPO in 100 mM NaCl full cells. A current of 2 mA cm^−2^ was used in all cases. For compound **11**, voltage versus discharge capacity data over five full charge–discharge cycles for 5 mM and 1 mM **11** full cells are overlaid. The 5 mM **11** in 100 mM NaCl and 10 mM 4-hydroxy-TEMPO in 100 mM NaCl full cell was cycled at a current of 1 mA cm^−2^. The 1 mM **11** in 100 mM NaCl and 2 mM 4-hydroxy-TEMPO in 100 mM NaCl full cell was cycled at a current of 0.2 mA cm^−2^. Cut-off voltages of 0.5 V (**10**, **11** and **13**), 1.90 V (**10**), 1.95 V (**11**) and 2.00 V (**13**) were used with 1 h potential holds being applied at the respective cut-off values. **d**, Discharge capacity versus cycle number for **11** at 10 mM, 5 mM and 1 mM concentrations. **e**, Normalized discharge capacity versus cycle number for **10**, **11** and **13** at 10 mM concentration.

**Extended Data Fig. 1:**
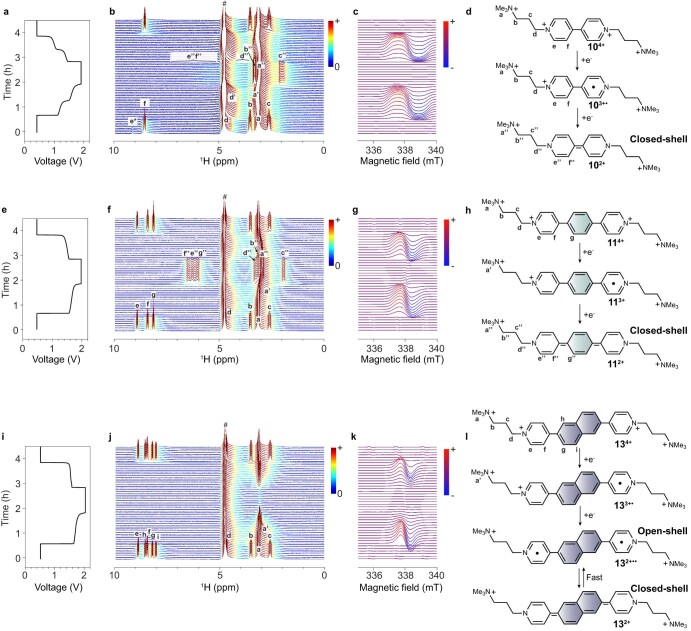
Coupled *operando* NMR and EPR spectroscopy. Voltage of a 10 mM (**a**) **10**, (**e**) **11**, (**i**) **13** in 100 mM NaCl and 20 mM 4-hydroxy-TEMPO in 100 mM NaCl full cell as a function of time for one full charge–discharge cycle. A current of 2 mA cm^−2^ was used. Cut-off voltages of 0.5 V (**10**, **11** & **13**), 1.90 V (**10**), 1.95 V (**11**) and 2.00 V (**13**) were used with 1 h potential holds being applied at the respective cut-off values. NMR **(b**,**f**,**j)** and EPR **(c**,**g**,**k**) spectra collected over the charge-discharge cycle. (**d**,**h**,**l**) Oxidation states of **10**, **11** and **13** and their respective NMR proton assignments. Chloride counterions are omitted for clarity. The proton assignment e* indicates that proton **e** undergoes fast hydrogen-deuterium exchange, reducing its intensity and limiting observation by NMR (section S7, Supplementary Information). The assignment # indicates overlap between the residual solvent signal and proton **d**. That no triplet diradical is directly observed by EPR for **13**^**2+**^ suggests that the *S* = 1 diradical is difficult to observe at low fields, there is fast singlet–triplet interconversion on experimental timescales, or there are low radical concentrations, either intrinsically or as a result of spin pairing of triplet diradicals^43^ to form EPR-silent dimers^44^.

**Extended Data Fig. 2:**
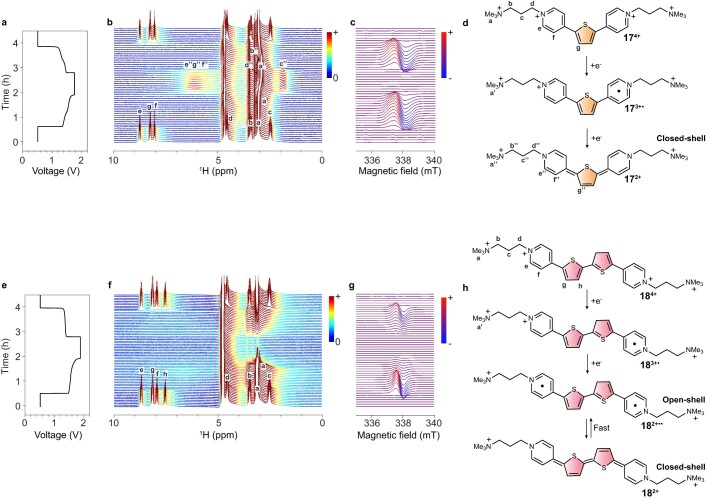
Coupled *operando* NMR and EPR spectroscopy for 17 and 18. Voltage of a 10 mM (**a**) **17**, (**e**) **18** in 100 mM NaCl and 20 mM 4-hydroxy-TEMPO in 100 mM NaCl full cell as a function of time for one full charge-discharge cycle. A current of 2 mA cm^−2^ was used. Cut-off voltages of 0.5 V (**17** & **18**), 1.75 V (**17**), and 1.85 V were used with 1 h potential holds being applied at the respective cut-off values. NMR **(b**,**f)** and EPR (**c**,**g**) spectra collected over the charge-discharge cycle. **(d**,**h**) Oxidation states of **17** and **18** and their respective NMR proton assignments. Chloride counterions are omitted for clarity.

**Extended Data Fig. 3:**
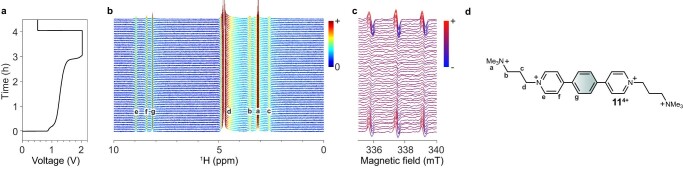
Coupled *operando* NMR and EPR spectroscopy for 11 at 1 mM. Voltage of a 1 mM (**a**) **11** in 100 mM NaCl and 2 mM 4-hydroxy-TEMPO in 100 mM NaCl full cell as a function of time for one full charge-discharge cycle. A current of 0.2 mA cm^−2^ was used. Cut-off voltages of 0.5 V and 1.95 V were used with 1 h potential holds being applied at the respective cut-off values. NMR (**b**) and EPR (**c**) spectra collected over the charge-discharge cycle. EPR spectral features indicate the presence of ultra-trace quantities of 4-OH-TEMPO crossover. (**d**) Structure of **11** and its respective NMR proton assignments.

Among compounds showing electrochemical reversibility, **10**, **11** and **13** feature wide, intermediate and narrow singlet–triplet gaps, respectively, increasing degrees of core conjugation (**10** < **11** < **13**) and both classical (**10**) and extended (**11** and **13**) cores. Compounds **17** and **18** can be regarded as heterocyclic analogues of **11** and **13**, respectively. As such, they constitute a representative subset of compounds for further analysis. To gain insights into the electrochemical behaviour of these compounds during cell operation, a series of coupled operando nuclear magnetic resonance (NMR) and electron paramagnetic resonance (EPR) studies were performed (Extended Data Figs. 1–3 and Extended Data Table 1). The NMR and EPR results were further used to calculate both comproportionation (*K*_c_) and dimerization (*K*_d_) equilibrium constants. The obtained *K*_c_ and *K*_d_ together with *E*_ST_ values (Extended Data Table 2 and Fig. 2a,b) provide insights into observed trends in capacity fade (Fig. 2d,e).

**Extended Data Fig. 4:**
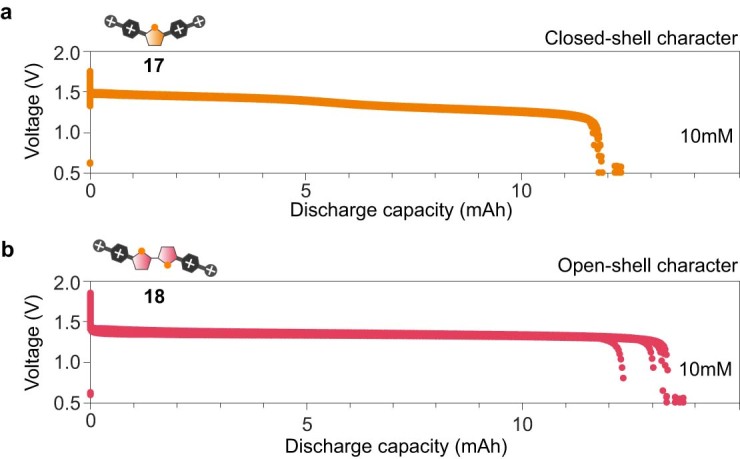
Performance characteristics of 17 and 18. Voltage *versus* discharge capacity over five full charge-discharge cycles for 10 mM (**a**) **17** and (**b**) **18** in 100 mM NaCl and 20 mM 4-hydroxy-TEMPO in 100 mM NaCl full cells. A current of 2 mA cm^−2^ was used in all cases. Cut-off voltages of 0.5 V (**17** & **18**), 1.75 V (**17**) and 1.85 V (**18**) were used with 1 h potential holds being applied at the respective cut-off values.

**Extended Data Fig. 5:**
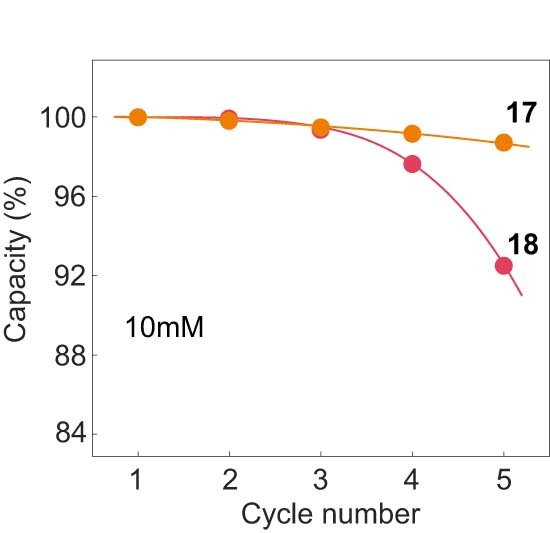
Normalized discharge capacity for 17, and 18 at 10 mM. Coulombic efficiencies for **17** and **18** were 78.6 ± 0.3% and 79.7 ± 2.8% respectively.

For all compounds, whereas Coulombic efficiencies fell within a narrow range (74–81%), values for capacity fade revealed a striking series of different trends. Considering processes at the molecular level, extended bispyridiniums with narrower *E*_ST_ values (**13**, **18**) showed significantly higher degrees of capacity fade relative to those with wider *E*_ST_ gaps (**11**, **17**) (Fig. 2c and Extended Data Figs. 4 and 5), suggesting that irreversible redox processes similar in nature to those observed by cyclic voltammetry for compounds **14**, **15**, **16** and **19** (Fig. 1e) probably take place. This is further tied with NMR and EPR findings (Extended Data Figs. 1 and 2), which show fundamentally distinct spectral characteristics for narrow versus wide *E*_ST_. Compounds **10**, **11** and **17** (wide *E*_ST_) show pronounced closed-shell character for doubly reduced species with electron spins paired intramolecularly (singlet), whereas **13** and **18** (narrow *E*_ST_) have thermaly accessible paramagnetic triplet states (diradical; unpaired spins) under the same conditions. These results provide evidence for the presence of two distinct regimes of electrochemical performance, delineated by *E*_ST_. From the supramolecular point of view, among compounds with a wider *E*_ST_ (**10**, **11**, **17**), those that show high monoradical concentrations (**10**; low *K*_d_ and limited electron pairing through intermolecular interactions) at all states of charge (SOC) also show greater degrees of capacity fade (Fig. 2a,c,e). Collectively, these results indicate that capacity fade in bispyridinium compounds is primarily linked to the formation of open-shell structures, either monoradicals or diradicals. Thus, processes that lower radical concentrations at all SOC (low *K*_c_, high *K*_d_ and high *E*_ST_) should correlate with improved capacity retention.

Low *K*_c_ and high *E*_ST_ processes give rise to closed-shell structures, whereas high *K*_d_ processes retain monoradicals as spin-paired *π-*dimers; at present, these are thought to contribute directly to capacity fade^1,4,7–11^. As *π*-dimerization is a concentration-dependent process favouring higher degrees of dimerization at higher monoradical concentrations, an extra set of RFB runs was carried out for compound **11** (that is, a high *K*_d_ compound) at concentrations of 1, 5 and 10 mM to determine the extent to which *π*-dimerization contributes to capacity fade (Fig. 2d). As the concentration of **11** increased, corresponding to higher degrees of *π*-dimerization, both capacity retention and Coulombic efficiencies increased. At 10 mM, Coulombic efficiencies of 77% were observed. These dropped slightly to 74% at 5 mM and then to 18% at 1 mM. Concomitant with this, at 10 mM capacity fade rates of 0.01% per cycle were obtained, while at 5 mM they rose sharply to 9.64% per cycle. At concentrations of 1 mM, consistent with low degrees of *π*-dimerization, however, a completely different set of charge–discharge characteristics was observed. Instead of charging processes corresponding to reduction of **11**, a new set of processes with an onset voltage of 0.82 V versus 4-hydroxy-TEMPO emerged (Extended Data Fig. 3). These processes proceeded over much longer timescales than those characteristic of bispyridinium charging and resulted in both an accumulation of hydroxide increasing pH from 7 to 12, and an almost complete loss of system capacity within the first charge–discharge cycle. Notably, during these experiments no evidence of compound **11** decomposition products was observed, suggesting that other processes than chemical degradation are accountable for the capacity fade.

## Association-mediated air tolerance of electrolytes

**Fig. 3:**
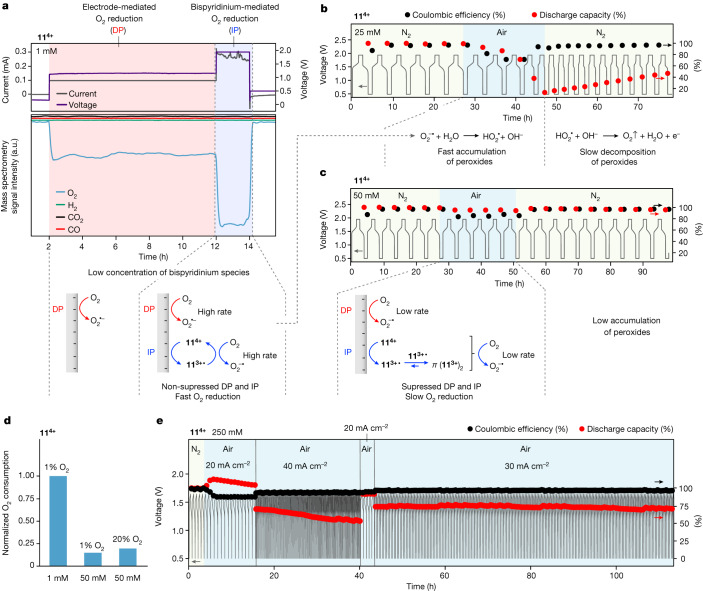
The influence of oxygen on bispyridinium redox processes and its suppression through *π*-dimerization. **a**, OEMS of a 1 mM **11** in 100 mM NaCl and 2 mM 4-hydroxy-TEMPO in 100 mM NaCl H cell during one full charge–discharge cycle in an atmosphere of 1% O_2_ in Ar. A current of 0.2 mA was used. A potential hold of 2 h was applied at 1.95 V after 8 h of charging. **b**, Voltage, normalized discharge capacity and Coulombic efficiency of a 25 mM **11** in 500 mM NaCl and 50 mM 4-hydroxy-TEMPO in 500 mM NaCl full cell cycled six times in N_2_, five times in air and ten times in N_2_. A current of 5 mA cm^−2^ was used. **c**, Voltage, normalized discharge capacity and Coulombic efficiency of a 50 mM **11** in 500 mM NaCl and 100 mM 4-hydroxy-TEMPO in 500 mM NaCl full cell cycled six times in N_2_, five times in air and ten times in N_2_. A current of 5 mA cm^−2^ was used. **d**, OEMS of 50 mM H cells subjected to a 2 h potential hold at 1.95 V under atmospheres of 1% O_2_ in Ar and 20% O_2_ in Ar, respectively, relative to that of the 1 mM H cell at 1.95 V described in **a**. A current of 1.55 mA was used in both cases. **e**, Voltage, normalized discharge capacity and Coulombic efficiency of a 250 mM **11** and 250 mM 4-hydroxy-TEMPO in 1 M NaCl full cell cycled five times in N_2_ at a current density of 20 mA cm^−2^, 15 times in air at a current density of 20 mA cm^−2^, 111 times in air at a current density of 40 mA cm^−2^, five times in air at a current density of 20 mA cm^−2^ and 200 times in air at a current density of 30 mA cm^−2^. Cut-off voltages of 0.5 V and 1.65 V. DP, direct pathway; IP, indirect pathway.

Online electrochemical mass spectrometry (OEMS)^5,32^ conducted on a 1 mM sample of **11** revealed a sharp steady-state decrease in oxygen partial pressure in the headspace above the electrolyte solution during charging under a continuous flow of 1% O_2_ in Ar (Fig. 3a). This oxygen consumption became substantially more pronounced once the bispyridinium reduction potential had been reached. At no stage during operation was any change detected in the hydrogen partial pressure, despite operating at cell voltages outside the thermodynamic stability window of water (1.23 V). Collectively, these results indicate that the increases in pH during cycling are tied to consumption of gaseous oxygen, not water splitting, and that reduced bispyridinium species facilitate this process. On this basis, the two-electron direct reduction of trace dissolved oxygen by means of the peroxide pathway to form hydroxide anions is proposed as a parasitic process (*E* = −0.065 V versus SHE; 0.87 V versus 4-hydroxy-TEMPO), for which **11**^**3+•**^ probably serves as a redox mediator^33–37^. At higher concentrations, typical bispyridinium redox behaviour was recovered with no further evidence for charge plateaus corresponding to other processes, but with similar increases in pH observed. This suggests that oxygen reduction still occurs. As (1) the onset of dimerization in bispyridinium species typically occurs around 0.1 mM in water^4^, (2) viologens are known redox mediators for oxygen reduction^33–37^ and (3) *π*-dimerization favours higher degrees of association at higher concentrations of corresponding monoradical^38^, it was proposed that higher degrees of *π*-dimerization, and hence *K*_d_, may play a role in mitigating competing side reactions between monoradical species and trace impurities such as oxygen during operation.

**Extended Data Fig. 6:**
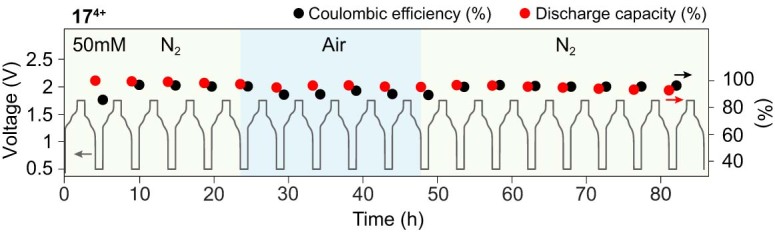
Air tolerance of 17. Voltage, normalised discharge capacity and Coulombic efficiency of a full cell comprising 50 mM **17** in 500 mM NaCl and 100 mM 4-hydroxy-TEMPO in 500 mM NaCl cycled 5 times in N_2_, 5 times in air, and 8 times in N_2_. A current of 5 mA cm^−2^ was used.

To confirm this, an extra set of experiments was carried out at 25 and 50 mM of compound **11** (Fig. 3b,c). At these concentrations, cells were galvanostatically cycled six times under N_2_, after which electrolyte solutions were exposed to air and cycled a further five times. Following this, solutions were sparged with N_2_ and cycled a final ten times under N_2_. In both cases, capacity was stable during initial runs under N_2_. However, on introduction of air, whereas capacity fell sharply over five cycles for the 25 mM case, it remained almost unchanged for the 50 mM case. In contrast to 50 mM, the 25 mM system required subsequent sparging with inert gas to regain lost capacity. Follow-up OEMS studies carried out on compound **11** at 50 mM with O_2_ partial pressures of 1 and 20% in Ar revealed oxygen consumption per mole of **11** to be substantially lower (more than five times) in comparison with the 1 mM system (Fig. 3c,d). Notably, the consumption of oxygen is independent of its Henry’s law partitioning in water, but rather depends on the concentration of bispyridinium species (Fig. 3d). This indicates that the reduction of oxygen is probably suppressed by both (1) faster interfacial electron transfer from the electrode to bispyridinium species than to oxygen (direct pathway), and (2) *π*-dimerization, which efficiently lowers the fraction of free radical monomeric species acting as electron mediators (indirect pathway) (Fig. 3a,c); this was corroborated by our DFT and CCSD(T) calculations (Supplementary Information Section 8). Indeed, rates of electron transfer from the electrode surface to compounds **11** and **17** (*k*_0,11_ = 1.98 × 10^−2^ cm s^−1^, *k*_0,17_ = 2.8 × 10^−3^ cm s^−1^)^22,23^ compare favourably with the analogous rate for direct electron transfer to oxygen (*k*_0,O2_ = 8.4 ×10^−4^ cm s^−1^)^39^. Thus, at sufficiently high concentrations the reduction of dissolved oxygen is effectively rendered negligible. Analogous results were obtained for a 50 mM **17** full cell (Extended Data Fig. 6), indicating that these phenomena are general.

**Extended Data Fig. 7:**
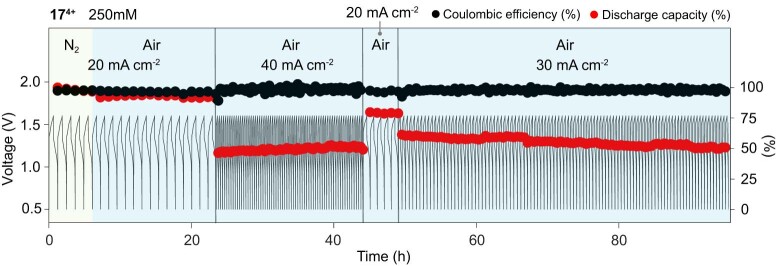
Extended cycling of 17 under air at 250 mM. Voltage, normalised discharge capacity and Coulombic efficiency of a 250 mM **17** and 250 mM 4-hydroxy-TEMPO in 1 M NaCl full cell cycled 5 times in N_2_ at a current density of 20 mA cm^−2^, 15 times in air at a current density of 20 mA cm^−2^, 67 times in air at a current density of 40 mA cm^−2^, 5 times in air at a current density of 20 mA cm^−2^, and 100 times in air at a current density of 30 mA cm^−2^. Cut-off voltages of 0.5 V and 1.60 V were used.

As a final demonstration under representative flow battery conditions, a full cell consisting of 250 mM **11** was cycled five times under N_2_ at 20 mA cm^−2^, 15 times under air at 20 mA cm^−2^, 111 times under air at 40 mA cm^−2^, five times under air at 20 mA cm^−2^ and a final 250 times under air at 30 mA cm^−2^ (Fig. 3e). An upper cut-off voltage of 1.65 V was applied to cycle the cell under one-electron operation to ensure that results could be directly tied to monoradicals and their corresponding *π*-dimers rather than doubly reduced species and their corresponding higher order aggregates (Extended Data Table 1). During the first 130 cycles in air, an initial jump in capacity of 12.3% was observed, after which a slow temporal fade followed, which increased slightly with current. Towards the end of this step, however, the fade slowed and in the following 255 cycles it stabilized, reaching values of 1.41% per day over 200 cycles (0.021% per cycle, Extended Data Table 3) at 30 mA cm^−2^. This low fade rate probably occurs as accumulation of peroxides is arrested by both the direct and indirect pathways at high concentrations (Fig. 3b,c). Furthermore, no substantial losses in capacity were observed when switching to and from 20 mA cm^−2^ even after 111 cycles at 40 mA cm^−2^ in between, demonstrating the ability of the system to handle variable power demands as required in practical RFB applications. Similar results were obtained for compound **17** showing the generality of these performance characteristics (Extended Data Fig. 7 and Extended Data Table 3). Collectively, these results not only shed new light on the previously overlooked role of *π*-dimerization in capacity fade mechanisms, but also provide simple methods both of achieving air stability and of recovering capacity once initially lost.

## Conclusions

In summary, we developed an extensive synthetic library of associative bispyridinium electrolytes from which we arrived at (1) a general physico-chemical descriptor for irreversibility, (2) two distinct regimes of electrochemical performance and (3) improved cycling and air stability mediated by *π*-dimerization. We demonstrate that electron transfer and reactivity of reduced bispyridinium species are controlled by intra- and intermolecular pairing of free radicals allowing operation in air. This is fully reflected in enhanced air tolerance of RFB systems comprising electrolytes with wide singlet–triplet energy gaps (intramolecular electron pairing), low comproportionation and high dimerization constants (intermolecular electron pairing). These results stand in direct contrast to the prevailing view that *π*-dimerization itself contributes to capacity fade. On account of these findings, a molecular design based on simple conjugated cores featuring Kekulé substitution patterns promoting both closed-shell electronic structures and enhanced tendency for *π*-dimerization in the reduced electrolyte states is proposed. These insights provide a set of general principles for continued improvement of not only pyridinium-based systems but RFB materials more broadly.

## Methods

### Materials

We purchased 4-pyridinylboronic acid (97%), potassium carbonate (anhydrous) and sodium chloride (analytical) from Fisher Scientific. 1,4-dibromobenzene (greater than 98%), 4,4′-dibromobiphenyl (98%), 2,5-dibromothiophene (96%), 1,4-dibromonapthalene (98%), 2,6-dibromonapthalene (97%), 2,7-dibromonapthalene (99%), 9,10-dibromoanthracene (98%), 2,6-dibromopyridine (98%), 5,5′-dibromo-2,2’-bithiophene (99%), (3-bromopropyl)trimethylammonium bromide(97%) from Sigma-Aldrich, tetrakis(triphenylphosphine) palladium(0) (99.8% (metals basis), Pd 9% min) and palladium on carbon (10 wt.%) were purchased from Sigma-Aldrich. *N*,*N*-dimethylformamide (DMF) (greater than 99%, anhydrous), dichloromethane (greater than 99%), acetonitrile (greater than 99%), diethyl ether (greater than 99%), hydrochloric acid (99%), 4,4′-bipyridine (99%), 4-hydroxy-2,2,6,6-tetramethylpiperidine-1-oxyl (97%) and deuterium oxide (99.9% atom% D) were purchased from Sigma-Aldrich. Milli-Q water was used for preparation of all non-deuterated aqueous solutions. Materials were used as obtained without further purification.

### Synthetic procedures

Bispyridine cores with a variety of redox modulators (**1**–**9**) and their fully alkylated analogues (**10**–**19**) were synthesized according to previously developed protocols, and modified as appropriate (Supplementary Information Section 3). General synthesis of compounds **1**–**9** was by means of Suzuki–Miyaura coupling^25^. 4-pyridinylboronic acid (1.25 g, 10 mmol), 1,4-dibromobenzene (1 g, 4.2 mmol) and potassium carbonate (2.8 g, 20.4 mmol) were added to a 7:1 mixture of degassed DMF and water (120 ml). Tetrakis(triphenylphosphine)palladium(0) (0.39 g, 0.34 mmol) was added to the reaction mixture and the solution heated to 100 °C under N_2_ for 72 h. Thereafter, the reaction mixture was cooled to room temperature and filtered. The organic phase was concentrated under vacuum, and the residue dissolved in CH_2_Cl_2_ (150 ml) and washed three times with water (50 ml each). Concentrated HCl was then added dropwise to the collected organic phase, resulting in precipitation of the product. The precipitate was collected by filtration and then dissolved in H_2_O. Finally, aqueous NaOH (10 M) was added dropwise to the H_2_O layer until the pH was roughly 8–9, resulting in the precipitation of the pure product (Supplementary Information Section 2).

General synthesis of compounds **10**–**19** was by means of the Anderson–Menshutkin reaction. (3-bromopropyl)trimethylammonium bromide (1.00 g, 3.83 mmol) was added to a stirred solution of bipyridine (1.29 mmol) in anhydrous and degassed DMF (50 ml). The reaction mixture was heated to 100 °C and stirred for 48 h. Thereafter, the reaction mixture was cooled to 0 °C and the resulting precipitate was filtered and washed with cold DMF (3 × 20 ml), MeCN (3 × 20 ml) and diethyl ether (3 × 20 ml) to obtain the pure product. To obtain the corresponding tetrachloride salts, isolated bispyridinium salts were loaded onto an ion exchanged column. Tetrachloride salts were collected using three portions of water (50 ml each). On concentration under vacuum, compounds **10**–**19** were obtained (Supplementary Information Section 2). To obtain purities in excess of 99.9%, suitable for electrochemical studies, **10**–**19** were triturated six times from water using acetone.

### Cyclic voltammetry

Cyclic voltammetry experiments were carried out at 25 °C in N_2_ purged 0.1 M NaCl aqueous solutions with a Metrohm Eco Chemie Autolab PGSTAT12 potentiostat, working on GPES v.4.9 software. A three-electrode configuration was used with a 3 or 1.6 mm glassy carbon working electrode, a platinum counter electrode and RE-5B Ag/AgCl BASI reference electrode. The glassy carbon electrode was polished before each measurement using a 0.05 µm alumina-H_2_O slurry on a polishing cloth. Cyclic voltammetry was performed at 1 mM concentrations of active materials using a scan rate of 20 mV s^−1^.

### Operando flow cell tests

#### Cell assembly

The flow battery was purchased from Scribner Associates. Ultrahigh-purity sealed graphite flow plates with serpentine flow patterns were used for both electrodes. Each electrode comprised carbon felt (SGL) with a 5 cm^2^ active area. An anion exchange membrane (120 µm thickness, less than 10 Å pore size, Selemion) was placed between the two electrodes. Polytetrafluoroethylene frames with a thickness of 3 mm were used to position the electrodes with Viton gaskets 0.7 mm in thickness on each side of the frames. The current collectors were gold-plated copper plates. Anodized aluminium end plates with reactant input and output ports were used. A Masterflex L/S peristaltic pump (Cole-Parmer) was used to circulate the electrolytes through the electrodes at a flow rate of 40 rpm (roughly 20 ml min^−1^). Custom-made glassware made from Pyrex with gas inlet, outlet, liquid inlet and outlet were used as electrolyte reservoirs^5,6^.

#### Operando experimental protocol

The setup consisted of a flow battery, two peristaltic pumps, an electrochemical cycler (SP−150, BioLogic SAS), a benchtop EPR (MS5000, Magnettech) and an NMR (300 MHz, Bruker Avance III) spectrometer. The battery and the EPR spectrometer were positioned outside the 5G line of the NMR magnet. The electrolyte was pumped through the flow battery, then flowed through the EPR and NMR magnets and finally back to the electrolyte reservoir. The direction of flow was from the bottom to the top of both magnets. Perfluoroalkoxy tubes (1/16 inch, roughly 1.6 mm) were used to connect the electrolyte reservoir, the battery and the EPR and NMR sampling tubes. In the anolyte reservoir, the flow cell used 30 ml of a 0.01 M viologen in 0.1 M NaCl deuterated aqueous solution unless otherwise stated. In the catholyte reservoir, the flow cell used 50 ml of a 0.02 M 4-hydroxy-TEMPO in 0.1 M NaCl deuterated aqueous solution unless otherwise stated. Both reservoirs were purged with N_2_, degassed for 1 h and then kept under active N_2_ flow during cycling. The flow cell was galvanostatically charged and discharged five times at room temperature on a portable electrochemical cycler at current of 10 mA. For variable concentration experiments, currents of 1, 5 and 10 mA were used for 1, 5 and 10 mM concentrations, respectively. Pseudo-2D NMR experiments were performed on the flowing electrolyte solution by direct excitation with a 90° radiofrequency pulse. Each NMR spectrum was acquired by collecting eight free induction decays with a recycle delay of 15 s. The pulse width for a 90° pulse was 27 μs at 30 W. All spectra were referenced to the water chemical shift at 4.79 ppm. For in situ pulsed field gradient (PFG) NMR studies, the flow was stopped during the acquisition period (12 min). Diffusion coefficients were measured using a PFG stimulated echo pulse sequence. NMR data were processed using TopSpin v.3.6.3 (Bruker). EPR data were processed using EasySpin v.5.2.30 (ref. ^42^). Electrochemical data were processed using EC-lab v.11.36 (BioLogic).

#### Operando NMR and EPR results for 10, 11 and 13

NMR and EPR spectra were acquired while a full cell consisting of a 10 mM viologen and 20 mM 4-hydroxy-TEMPO both in D_2_O was galvanostatically cycled for five full charge–discharge cycles, the second of which is shown in Extended Data Fig. 1. On charging, the full cell containing **10** showed plateaus corresponding to two well-separated single-electron redox events. For **11** and **13**, redox events were sufficiently narrowly spaced such that single charge and discharge plateaus were observed in each case. Starting at 0.50 V versus 4-hydroxy-TEMPO, protons assigned to both the aliphatic and aromatic parts of the unreduced **10**^**4+**^, **11**^**4+**^ and **13**^**4+**^ ions were visible by NMR, with low degrees of signal intensity for proton **e** of **10**^**4+**^ occurring as a result of near-complete hydrogen-deuterium exchange within the first charge–discharge cycle (Supplementary Information Section 7). Above 0.50 V, all signals with the exception of **a** (the terminal quaternary amine protons) disappeared almost immediately with the concomitant emergence of EPR resonances, which were assigned to the radicals **10**^**3+•**^, **11**^**3+•**^ and **13**^**3+•**^.

At high SOC (1.90, 1.95 or 2.00 V for **10**, **11** or **13**, respectively), the potential was held constant for 1 h. During this period, new resonances appeared in the NMR spectra for **10** and **11** that were shifted substantially to lower frequencies relative to those observed at low SOC. These features were assigned to the diamagnetic, doubly reduced species **10**^**2+**^ and **11**^**2+**^. For **10**^**2+**^, substantial broadening of all resonances was observed, suggesting the presence of residual levels of radicals that exist in equilibrium with closed-shell ion **10**^**2+**^. For **13**, however, an ion with an extended core, but with a calculated narrower singlet–triplet gap, no new resonances were observed during the potential hold. Instead, all NMR resonances were broadened substantially, including **a”**. Furthermore, no radical species were observed by EPR. As the *E*_ST_ for **13** is small, population of paramagnetic thermally accessible triplet states may be responsible for the notable NMR line broadening observed. That no triplet diradical is directly observed by EPR suggests that the *S* = 1 diradical is difficult to observe at low fields, there is fast singlet–triplet interconversion on experimental timescales or there are low radical concentrations, either intrinsically or as a result of spin pairing of triplet diradicals^43^ to form EPR-silent dimers.

With structural assignments made for **10**, **11** and **13** at all SOC, an analysis of their associated redox equilibria was carried out. Expressing the two single-electron reductions as a comproportionation equilibrium^44^ with an equilibrium constant of comproportionation, *K*_c_, an equation for radical fraction as a function of battery SOC was obtained (Supplementary Information Section 6)^5,6^. This equation was then used to fit experimental radical fraction data (Fig. 2a), obtained by the EPR-based method of spin counting (Supplementary Information Section 5), from which the observed comproportionation equilibrium constant *K*_c,obs_ was extracted. On fitting, *K*_c,obs_ values of 0.75, 0.021 and 0.0021 were found for **10**, **11** and **13**, respectively (Extended Data Table 2). However, fits were modest at best and deviated substantially from experimental data especially for compound **13**, suggesting the presence of other phenomena not accounted for by the simple comproportionation model. To overcome this, cyclic voltammetry curve fitting^5,45^ was instead pursued from which much better agreement with experimental data was obtained (Supplementary Information Section 6). Relating the difference in first and second redox events obtained by cyclic voltammetry curve fitting to *K*_c_, ‘true’ *K*_c_ values could be estimated (Supplementary Information Section 6 and Extended Data Table 2) and compared to *K*_c,obs_ values. Respective *K*_c_ values for **10**, **11** and **13** were 3.2  × 10^5^, 1.3 and 2.9—substantially higher than the *K*_c,obs_ values. This discrepancy indicates that besides comproportionation, more reaction equilibria probably exist that result in lower observed radical fractions. The strongest candidates for these are dimerization equilibria^20^.

Whereas the presence of dimers could not be determined directly (as a result of both high radical concentrations affecting observation by NMR and the known EPR silence of viologen *π*-dimers^44^ affecting observation by EPR), in situ PFG NMR experiments carried out for **10**, **11** and **13** at 0%, 50% and 100% SOC (Extended Data Table 1) indicated a general decrease in diffusivity (*D*) from 0% to 100% SOC, especially for compounds **11** and **13** that show low radical concentrations at all SOC. These results indicate an increase in size of the cations on monoradical generation, consistent with the formation of dimers.

### Ultraviolet–visible light spectroelectrochemical studies

To gain further insights into the nature of monoradical dimerization, ex situ ultraviolet–visible light (UV–vis) spectroelectrochemical studies were carried out for **10**, **11** and **13**. Degassed solutions of **10**^**4+**^ (0.5 mM), **11**^**4+**^ (0.5 mM) and **13**^**4+**^ (0.5 mM) with were prepared using the Schlenk technique. The solutions were transferred into quartz cuvettes (10 mm path length) under N_2_ and the cuvettes and kept under a positive flow of N_2_. The samples were electrochemically reduced using a carbon paper working electrode and a gold counter electrode. Spectral data were acquired using a UV–vis spectrometer (Horiba, Duetta) immediately after the solutions were transferred into the cuvettes and electrochemically reduced.

On reduction, bands corresponding to singly reduced monoradical species and their *π*-dimers were observed (Fig. 2b)^40,41^, suggesting that *π*-dimerization is at least partially responsible for the simultaneously decreased radical concentrations and diffusivities observed in situ and ex situ. Accounting for this extra *π*-dimerization equilibrium, with associated equilibrium constant *K*_d_, using cyclic voltammetry-derived *K*_c_ values and fitting to the respective radical concentrations at 50% SOC^6^, *K*_d_ values of 0.29, 11 and 76 mM^−1^ were calculated for **10**, **11** and **13**, respectively (Extended Data Table 2 and Supplementary Information Section 6). As *π*-dimers are the lowest energy dimeric species known for viologens (Supplementary Fig. 1) they are expected to play more of a role in mediating bispyridinium electrolyte equilibria relative to other dimeric species such as *σ*-dimers and charge transfer complexes, which are frequently short lived^20^. Thus, during charging, *π*-dimerization equilibrium is shifted to the left for compound **10**, favouring accumulation of monoradical species and accounting for the similar diffusivities obtained at 0% and 50% SOC. For **11** and **13**, however, *π*-dimerization equilibrium is shifted to the right, favouring dimer formation and accounting for the significantly lower diffusivities obtained at 50% SOC relative to those at 0% SOC.

### Operando mass spectrometry

OEMS experiments were performed using a custom-made H cell, connected to gas flow system previously described using 1% and 20% O_2_ in Ar at 1.2 bar(a)^5,32^.

### Galvanostatic cycling flow cell studies at 25 and 50 mM

Flow cells were assembled as described above. For the 25 mM case, full cells were assembled from 25 mM **11** in 500 mM NaCl (30 ml) and 50 mM 4-hydroxy-TEMPO in 500 mM NaCl (50 ml). For the 50 mM case, full cells were assembled from 50 mM **11** in 500 mM NaCl (15 ml) and 100 mM 4-hydroxy-TEMPO in 500 mM NaCl (25 ml). A current of 5 mA cm^−2^ was used in both cases. Both reservoirs were purged with N_2_, degassed for 1 h and then kept under active N_2_ flow during cycling. The flow cell was galvanostatically charged and discharged at room temperature using a portable potentiostat. The cycling sequence consisted of six full charge and discharge cycles, after which the N_2_ was disconnected and the reservoirs opened to air. After 1 h, cells were cycled a further five times in air, at which point the reservoirs were closed, purged with N_2_ for 1 h and kept under a positive N_2_ flow for ten subsequent cycles under N_2_. Electrochemical data were processed using EC-lab 11.36 (BioLogic).

### Extended galvanostatic cycling flow cell studies at 250 mM

Flow cells were assembled as described above. Full cells were assembled from 250 mM **11** or **17** (10.0 ml for **11**, 12.5 ml for **17**) and 250 mM 4-hydroxy-TEMPO in 1 M NaCl (50 ml). Currents of 20 mA cm^−2^ (at a flow rate of 40 rpm), 30 mA cm^−2^ (at a flow rate of 60 rpm) and 40 mA cm^−2^ (at a flow rate of 80 rpm) were used in both cases. Both reservoirs were purged with N_2_, degassed for 1 h and then kept under active N_2_ flow during cycling for the first five cycles, after which the nitrogen flow was disconnected and the reservoirs opened to air. The flow cells were galvanostatically charged and discharged at room temperature using a portable potentiostat. Electrochemical data were processed using EC-lab 11.36 (BioLogic).

## Online content

Any methods, additional references, Nature Portfolio reporting summaries, source data, extended data, supplementary information, acknowledgements, peer review information; details of author contributions and competing interests; and statements of data and code availability are available at 10.1038/s41586-023-06664-7.

### Supplementary information

Supplementary Information Supplementary Sections 1–9, Tables 1–11 and Figs. 1–17.

## Figures and Tables

**Extended Data Table 1:**
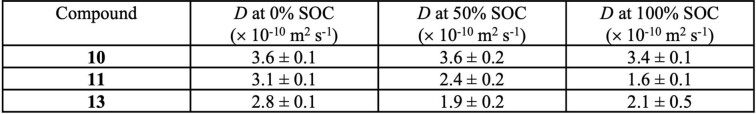
Summary of bispyridinium diffusivities at various SOC

**Extended Data Table 2:**
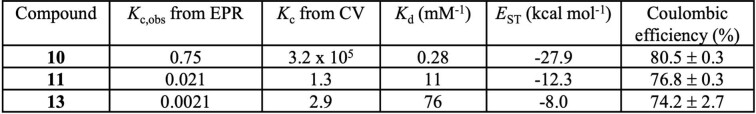
Summary of bispyridinium equilibrium data and Coulombic efficiencies

**Extended Data Table 3:**
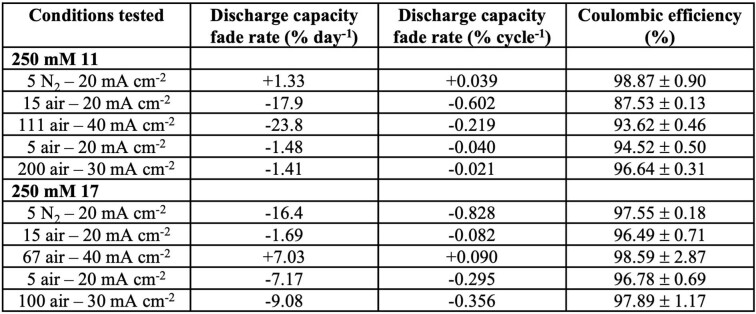
Summary of extended galvanostatic cycling performance for 11 and 17

## Data Availability

The experimental dataset generated and/or analysed during the current study is available from the corresponding authors on request.

## Code availability

Any code used during analysis is available from the corresponding authors on request.
