# Supplementary material for: Associative pyridinium electrolytes for air-tolerant redox flow batteries
Source: Nature. 2023 Nov 29;623(7989):949–55. doi: 10.1038/s41586-023-06664-7 (PMC10686829; doi:10.1038/s41586-023-06664-7)
Supplement: Supplementary file 1 — Supplementary Sections 1–9, Tables 1–11 and Figs. 1–17. [file 41586_2023_6664_MOESM1_ESM.pdf]

nature portfolio

https://doi.org/10.1038/s41586-023-06664-7

Supplementary information

Associative pyridinium electrolytes for air-tolerant redox flow batteries

In the format provided by the authors and unedited

Supplementary Information

Associative pyridinium electrolytes for air-tolerant redox flow batteries

*Mark E. Carrington[a,b^], Kamil Sokołowski[a,b^], Erlendur Jónsson[a], Evan Wenbo Zhao[a,c], Anton M. Graf[a,b], Israel Temprano[a], Jade A. McCune[a,b], Clare P. Grey[a *], Oren A. Scherman[a,b *]*

[a]*Yusuf Hamied Department of Chemistry, University of Cambridge, Cambridge, UK*
[b]*Melville Laboratory for Polymer Synthesis, Yusuf Hamied Department of Chemistry, University of Cambridge, Cambridge, UK*
[c]*Present address: Magnetic Resonance Research Center, Institute for Molecules and Materials, Faculty of Science, Radboud University Nijmegen, Nijmegen, NL*
[^]*these authors contributed equally*
[*]*E-mail: cpg27@cam.ac.uk, oas23@cam.ac.uk*

Table of contents

S1. Structures of reduced viologens
S2. Data for synthetic procedures
S3. Synthesis of bipyridines using palladium on carbon
S4. Computational screens of redox properties
S5. Radical concentration calculations
S6. Equilibrium models and fits to experimental data
S7. Evidence for other associated structures
S8. Thermodynamics of dimerization and stability to dioxygen
S9. References

S1. Structures of reduced viologens

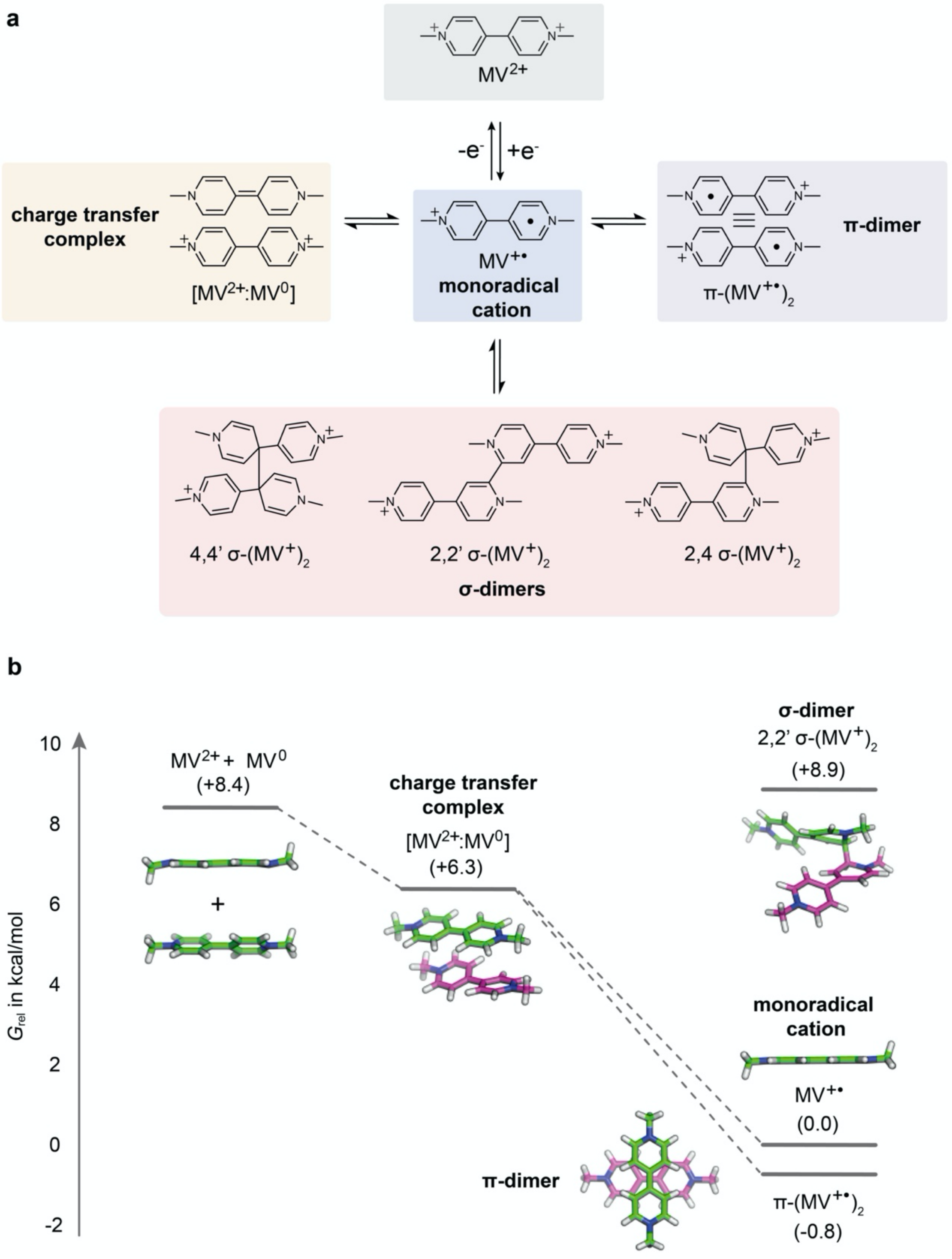

Supplementary Figure 1 | Redox chemistry and relative Gibb's Free Energies of methyl viologen and its reduced forms. (a) Redox chemistry of methyl viologen and association of its reduced forms. **(b)** Relative free energies of methyl viologen species and their associated forms on different oxidation states. Data are calculated using DFT-based methods as previously reported.[1]

S2. Data for synthetic procedures

Bipyridine cores with a variety of redox modulators (**1-9**) and their fully alkylated analogues (**10-19**) were synthesized according to previously developed protocols with modifications as appropriate.[2-8]

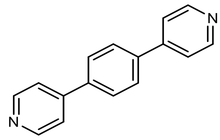

1: ^{1}H NMR (400 MHz, $CDCl_3$) δ [ppm]: 8.70 (dd, J = 4.4, 1.6 Hz, 4H), 7.77 (s, 4H), 7.56 (dd, J = 4.4, 1.6 Hz, 4H).

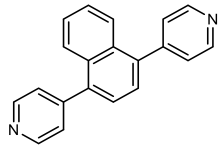

2: ^{1}H NMR (400 MHz, $CDCl_3$) δ [ppm]: 8.77 (dd, J = 4.4, 1.6 Hz, 4H), 7.92 (dd, J = 6.4, 3.2 Hz, 2H), 7.52 (dd, J = 6.4, 3.2 Hz; 2H), 7.49 (s, 2H), 7.48 (dd, J = 4.4, 1.6 Hz, 4H).

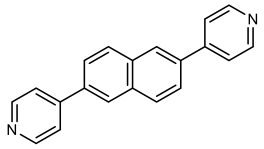

3: ^{1}H NMR (400 MHz, $CDCl_3$) δ [ppm]: 8.73 (dd, J = 4.4, 1.6 Hz, 4H), 8.16 (s, 2H), 8.05 (d, J = 8.5 Hz, 2H), 7.83 (d, J = 8.5 Hz, 2H), 7.66 (dd, J = 4.4, 1.6 Hz, 4H).

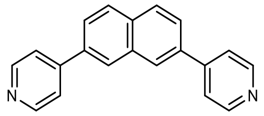

4: ^{1}H NMR (400 MHz, $CDCl_3$) δ [ppm]: 8.74 (dd, J = 4.4, 1.6 Hz, 4H), 8.21 (dd, J = 1.6, 0.8 Hz, 2H), 8.02 (d, J = 8.7 Hz, 2H), 7.82 (dd, J = 8.6, 1.8 Hz, 2H), 7.66 (dd, J = 4.4, 1.6 Hz, 4H).

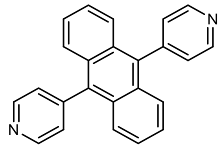

5: ^{1}H NMR (400 MHz, $CDCl_3$) δ [ppm]: 8.89 (dd, J = 4.0, 1.6 Hz, 4H), 7.62 (dd, J = 6.8, 3.2 Hz, 4H), 7.45 (dd, J = 4.0, 1.6 Hz, 4H), 7.40 (dd, J = 6.9, 3.2 Hz, 4H).

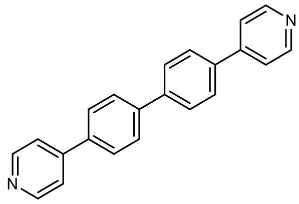

6: ¹H NMR (400 MHz, $CDCl_3$) δ [ppm]: 8.70 (dd, J = 4.4, 1.6 Hz, 4H), 7.77 (s, 8H), 7.57 (dd, J = 4.4, 1.6 Hz, 4H).

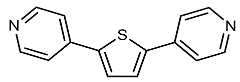

7: ¹H NMR (400 MHz, $CDCl_3$) δ [ppm]: 8.64 (dd, J = 4.4, 1.6 Hz, 4H), 7.55 (s, 2H), 7.51 (dd, J = 4.4, 1.6 Hz, 4H).

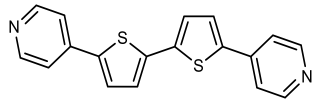

8: ¹H NMR (400 MHz, $CDCl_3$) δ [ppm]: 8.62 (dd, J = 4.4, 1.6 Hz, 4H), 7.48-7.44 (m, 6H), 7.25 (s, 2H).

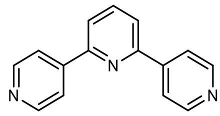

9: ¹H NMR (400 MHz, $CDCl_3$) δ [ppm]: 8.79 (dd, J = 4.0, 1.6 Hz, 4H), 8.08 (dd, J = 4.0, 1.6 Hz, 4H), 7.99 (dd, J = 7.5 Hz, 7.0 Hz, 2H), 7.92 (t, J = 6.0 Hz 1H).

Data for previously reported bispyridinium compounds **10 (refs. 9, 10)**, **11 (ref. 10) and 17 (ref. 11)** (bromide counterions omitted for clarity):

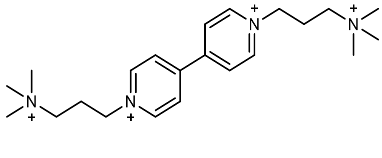

10: ¹H NMR (400 MHz, D_2O) δ [ppm]: 9.22 (d, J = 6.0 Hz, 4H), 8.65 (d, J = 6.0 Hz, 4H), 4.88 (t, J = 7.6 Hz, 4H), 3.63 – 3.59 (m, 4H), 3.22 (s, 18H), 2.75 – 2.67 (m, 4H).

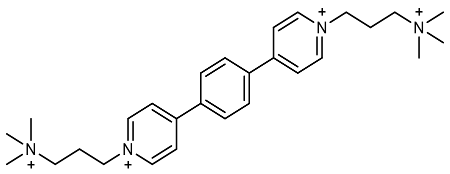

11: **^{1}H NMR** (400 MHz, D_2O) δ [ppm]: 8.99 (d, J = 7.0 Hz, 4H), 8.49 (d, J = 6.9 Hz, 4H), 8.21 (s, 4H), 4.83 – 4.75 (m, 4H), 3.62 – 3.57 (m, 4H), 3.22 (s, 18H), 2.73 – 2.63 (m, 4H).

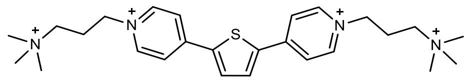

17: **^{1}H NMR (**400 MHz, D_2O) δ [ppm]: 8.89 (d, J = 6.4 Hz, 4H), 8.39 (d, J = 6.0 Hz, 4H), 8.19 (s, 2H), 4.73 (t, J = 7.7 Hz, 4H), 3.66 – 3.50 (m, 4H), 3.21 (s, 18H), 2.73 – 2.60 (m, 4H).

Data for previously reported bispyridinium compounds **12, 13 and 18 (all from ref. 6)** (bromide counterions omitted for clarity):

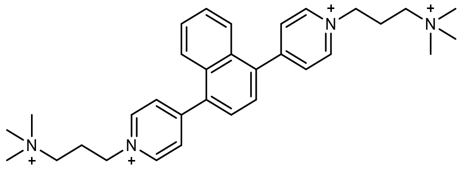

12: **^{1}H NMR** (400 MHz, D_2O) δ [ppm]: 9.08 (d, J = 5.2 Hz, 4H), 8.39 (d, J = 5.0 Hz, 4H), 8.04 – 8.01 (m, 2H), 7.85 (s, 2H), 7.79 – 7.75 (m, 2H), 4.87 (t, J = 8.0 Hz, 4H), 3.66 – 3.62 (m, 4H), 3.24 (s, 18H), 2.78 – 2.70 (m, 4H). **^{13}C NMR** (100 MHz, D_2O) δ [ppm]: 157.70, 144.26, 136.31, 130.01, 129.52, 128.42, 127.62, 125.08, 62.47, 57.65, 53.20, 53.16, 53.13, 24.60. **MS** ESI-MS: m/z $[M]^{4+}$ calc for $C_{32}H_{44}N_4$: 121.0886, found: 121.0888. **FTIR** ν [cm^{-1}]: 667, 742, 768, 832, 845, 878, 912, 929, 963, 1063, 1120, 1190, 1232, 1313, 1361, 1393, 1426, 1472, 1520, 1558, 1635, 3020, 3370. 80% yield.

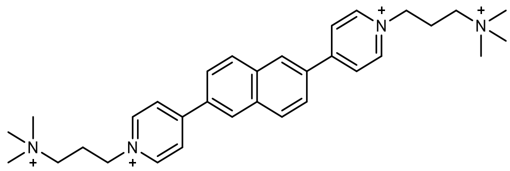

13: **^{1}H NMR** (400 MHz, D_2O) δ [ppm]: 8.97 (d, J = 5.0 Hz, 4H), 8.69 (s, 2H), 8.56 (d, J = 5.2 Hz, 4H), 8.36 (d, J = 8.0 Hz, 2H), 8.16 (d, J = 8.0 Hz, 2H), 4.79 – 4.76 (m, 4H), 3.61 – 3.57 (m, 4H), 3.24 (s, 18H), 2.78 – 2.70 (m, 4H). **^{13}C NMR** (100 MHz, D_2O) δ [ppm]: 156.27, 144.27, 134.06, 133.06, 130.73, 128.82, 125.65, 125.23, 62.46, 57.17, 53.18, 24.56. **MS** ESI-MS: m/z $[M]^{4+}$ calc for $C_{32}H_{44}N_4$: 121.0886, found: 121.0885. **FTIR** ν [cm^{-1}]: 741, 768, 833, 846, 887, 930, 964, 1039, 1065, 1120, 1148, 1190, 1233, 1313, 1361, 1392, 1424, 1472, 1520, 1558, 1636, 1711, 3021, 3367. 89% yield.

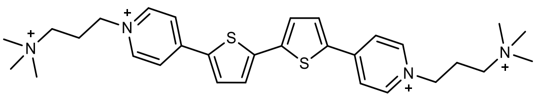

18: **^{1}H NMR (**400 MHz, D_2O) δ [ppm]: 8.76 (d, J = 6.8 Hz, 4H), 8.26 (d, J = 6.0 Hz, 4H), 8.07 (d, J = 3.6 Hz, 2H), 7.70 (d, J = 3.6 Hz, 2H), 4.66 (t, J = 7.8 Hz, 4H), 3.57 – 3.53 (m, 4H), 3.20 (s, 18H), 2.65 – 2.57 (m, 4H). **^{13}C NMR** (100 MHz, D_2O) δ [ppm]: 148.90, 143.94, 142.97, 137.00, 133.22, 128.33, 122.70, 62.47, 56.82, 53.14, 53.11, 53.08, 24.41. **MS** ESI-MS: m/z $[M]^{4+}$ calc for $C_{30}H_{42}N_4S_2$: 130.5707, found: 130.5706. **FTIR** ν [cm^{-1}]: 667, 728, 741, 772, 805, 845, 875, 942, 964, 1000, 1048, 1071, 1084, 1118, 1174, 1204, 1225, 1240, 1307, 1330, 1357, 1381, 1407, 1433, 1465, 1492, 1528, 1553, 1628, 3002, 3016, 3041, 3423. 82% yield.

Data for newly synthesized bispyridinium compounds **14-16,** & **19** (bromide counterions omitted for clarity) based on previously reported methodologies for synthesis of bipyridine cores[2-8] and alkylation with TMAP groups[6, 9-11]:

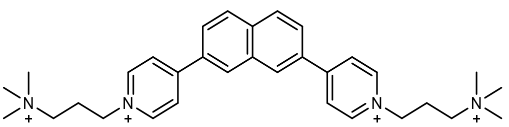

14: **^{1}H NMR** (400 MHz, D_2O) δ [ppm]: 8.98 (d, J = 5.6 Hz, 4H), 8.75 (s, 2H), 8.56 (d, J = 6.2 Hz, 4H), 8.28 (d, J = 8.8 Hz, 2H), 8.18 (d, J = 8.8 Hz, 2H), 4.79 – 4.76 (m, 4H), 3.61 – 3.59 (m, 4H), 3.22 (s, 18H), 2.72 – 2.64 (m, 4H). **^{13}C NMR** (100 MHz, D_2O) δ [ppm]: 156.42, 144.23, 135.64, 132.72, 132.24, 130.21, 129.54, 126.46, 125.63, 62.51, 57.20, 53.15, 24.54. **MS** ESI-MS: m/z $[M]^{4+}$ calc for $C_{32}H_{44}N_4$: 121.0886, found: 121.0887. **FTIR** ν [cm^{-1}]: 848, 923, 940, 968, 1057, 1066, 1175, 1241, 1349, 1394, 1409, 1477, 1532, 1559, 1623, 1638, 2901, 2988, 3351, 3661. 85% yield.

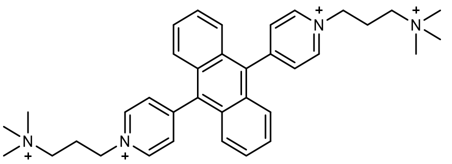

15: **^{1}H NMR** (400 MHz, D_2O) δ [ppm]: 9.23 (d, J = 9.2 Hz, 4H), 8.38 (d, J = 6.0 Hz, 4H), 7.68 – 7.61 (m, 8H), 4.96 (t, J = 7.2 Hz, 4H), 3.73 – 3.69 (m, 4H), 3.28 (s, 18H), 2.87 – 2.78 (m, 4H). **^{13}C NMR** (100 MHz, D_2O) δ [ppm]: 157.54, 144.69, 132.48, 131.46, 128.06, 127.43, 125.31, 62.56, 57.98, 53.22, 24.72. **MS** ESI-MS: m/z $[M]^{4+}$ calc for $C_{36}H_{46}N_4$: 133.5925, found: 133.5925. **FTIR** ν [cm^{-1}]: 679, 734, 819, 831, 893, 923, 960, 1032, 1109, 1139, 1160, 1183, 1216, 1292, 1341, 1394, 1418, 1448, 1477, 1519, 1559, 1640, 3059, 3121, 3352. 83% yield.

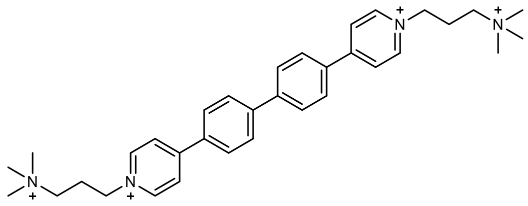

16: ^{1}H NMR (400 MHz, D_2O) δ [ppm]: 8.92 (d, J = 6.0 Hz, 4H), 8.46 (d, J = 5.6 Hz, 4H), 8.16 (d, J = 7.6 Hz, 4H), 8.07 (d, J = 7.2 Hz, 4H), 4.75 (t, J = 7.2 Hz, 4H), 3.59 – 3.56 (m, 4H), 3.21 (s, 18H), 2.70 – 2.62 (m, 4H). **^{13}C NMR** (100 MHz, D_2O) δ [ppm]: 156.41, 144.16, 142.75, 133.38, 128.80, 128.24, 125.20, 62.48, 57.10, 53.08, 24.47. **MS** ESI-MS: m/z $[M]^{4+}$ calc for $C_{34}H_{46}N_4$: 127.5925, found: 127.5925. **FTIR** ν [cm^{-1}]: 739, 756, 817, 836, 872, 927, 964, 1057, 1066, 1187, 1201, 1231, 1295, 1394, 1404, 1469, 1492, 1525, 1543, 1570, 1603, 1635, 2901, 2989, 3363, 3662. 81% yield.

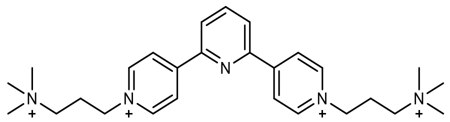

19: ^{1}H NMR (400 MHz, D_2O) δ [ppm]: 9.08 (d, J = 5.4 Hz, 4H), 8.90 (d, J = 5.2 Hz, 4H), 8.48 (d, J = 8.0 Hz, 2H), 8.37 (t, 7.5 Hz, 1H), 4.82 (t, J = 7.6 Hz, 4H), 3.62 – 3.58 (m, 4H), 3.21 (s, 18H), 2.73 – 2.65 (m, 4H). **^{13}C NMR** (100 MHz, D_2O) δ [ppm]: 153.88, 150.95, 144.96, 140.36, 125.77, 125.64, 62.54, 57.66, 53.24, 24.64. **MS** ESI-MS: m/z $[M]^{4+}$ calc for $C_{27}H_{41}N_5$: 108.8335, found: 108.8337. **FTIR** ν [cm^{-1}]: 722, 811, 871, 925, 962, 991, 1066, 1099, 1183, 1230, 1293, 1349, 1408, 1451, 1477, 1520, 1568, 1590, 1638, 2494, 2989, 3363, 3671. 76% yield.

S3. Synthesis of bipyridines using palladium on activated carbon

To demonstrate the potential for scalability in the synthesis of extended bispyridinium compounds, the use of palladium on activated carbon, an industrial hydrogenation catalyst, was employed rather than tetrakis(triphenylphosphine)palladium(0). Synthesis proceeded under unoptimized conditions as follows:

4-pyridinylboronic acid (1.72 g, 14 mmol), 1,4-dibromobenzene (1.00 g, 4.2 mmol) and potassium carbonate (3.52 g, 25 mmol) were added to a 1:1 mixture of degassed DMF and water (120 ml). Palladium on activated carbon (100 mg) was added to the reaction mixture and the solution heated to 100 °C under N_2 for 72 h. Thereafter, the reaction mixture was cooled to room temperature and filtered. The organic phase was concentrated under vacuum and the residue dissolved in CH_2Cl_2 (150 mL) and washed three times with water (50 mL each). Concentrated HCl was then added dropwise to the collected organic phase, resulting in precipitation of the product. The precipitate was collected by filtration and then dissolved in H_2O. Finally, aqueous NaOH (10 M) was added dropwise to the H_2O layer until the pH was *ca.* 8-9, resulting in the precipitation of the pure product (89 mg, 9%).

While under unoptimized conditions yields were low, ^{1}H NMR spectra of **1** revealed product purities in excess of 99% – higher than those obtained using tetrakis(triphenylphosphine)palladium(0) (Supplementary Fig. 2). As palladium on activated

carbon is (i) a well-established industrial heterogeneous catalyst, (ii) is substantially cheaper than tetrakis(triphenylphosphine)palladium(0), and (iii) does not possess contaminating ligands that need to be separated from the reaction product giving rise to higher purities, it is anticipated that such a catalyst, under optimized conditions, can lower the cost of synthesis dramatically and improve scalability of production.

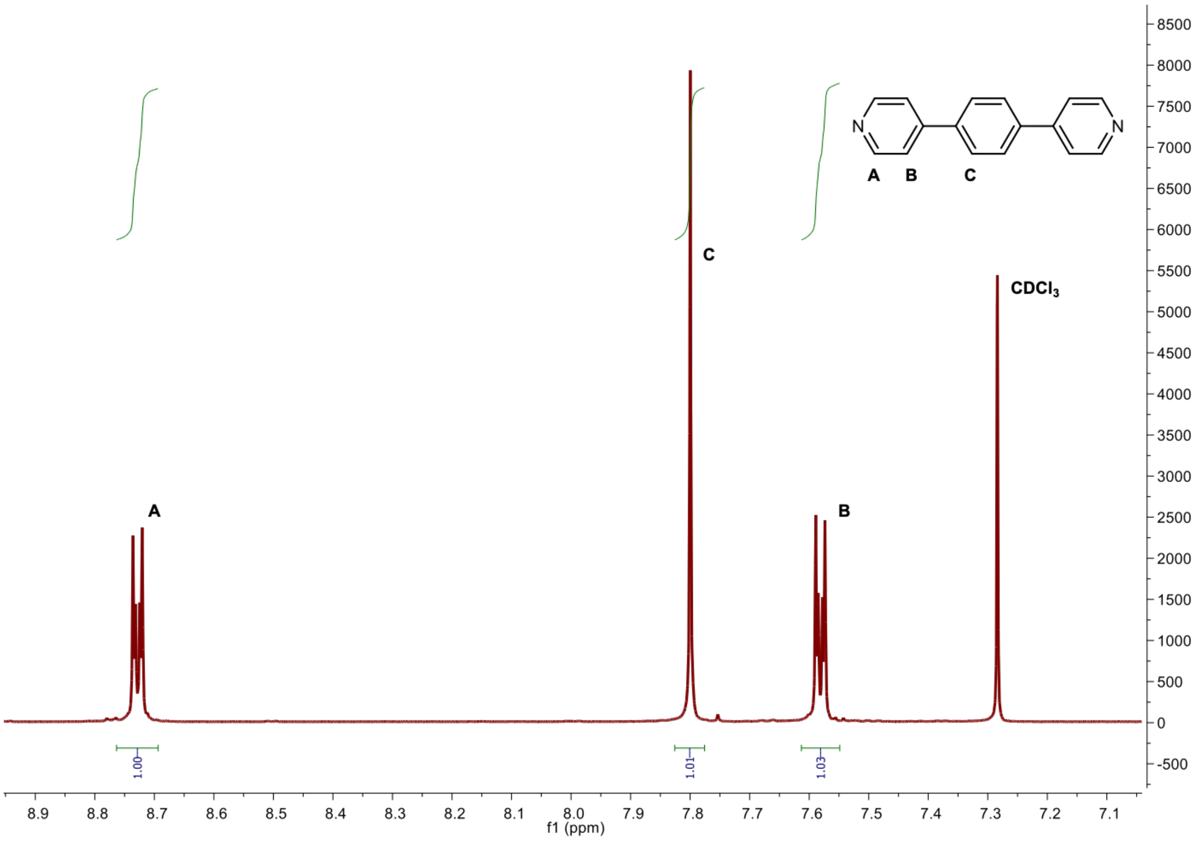

Supplementary Figure 2 | ^{1}H NMR spectrum of 1 synthesized using palladium on activated carbon as a catalyst.

To determine the extent to which palladium on activated carbon can serve as a recyclable catalyst, leading to further cost reductions for bipyridine synthesis, a series of reaction cycles were carried out wherein the palladium on carbon catalyst used in one batch was filtered off and used in the subsequent batch. To determine the optimal reaction length for carrying out such catalyst recycling studies, a kinetic investigation was first carried out. For this investigation, the reaction was run as before, but with aliquots being taken at regular time points. The results are shown in Supplementary Fig. 3. Until 68 h, the reaction proceeded cleanly with a distribution of mono and di-arylated products. Above 68 h, decomposition products started to form providing a suitable end-point for subsequent cycling studies. The yield after 118 h was 8%.

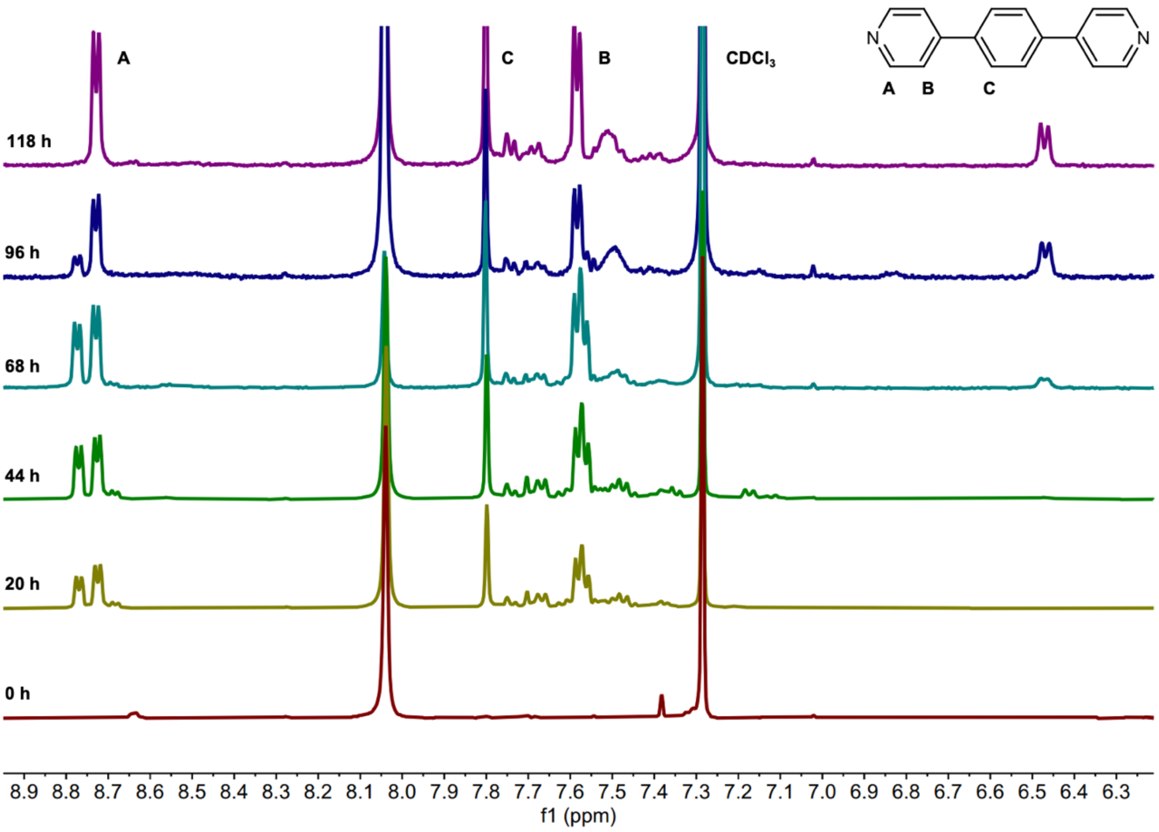

Supplementary Figure 3 | ^{1}H NMR spectra of aliquots taken from the crude reaction mixture at various points during synthesis.

Cycling studies were then carried out, following a similar procedure to that described above, but using the same batch of catalyst for each run. Over five full reaction cycles, each run for 68 h, the following results were obtained:

run 1: 9% yield, run 2: 5% yield, run 3: 3% yield, run 4: 1% yield, run 5: 0% yield

These results demonstrate catalyst recyclability, albeit with diminishing degrees of catalytic activity from cycle to cycle. Future work is needed to optimize catalytic conditions and to determine whether activity can be recovered once initially lost. Collectively, however, these results provide a synthetic pathway through which bipyridine compounds can be produced cheaply at larger scales.

S4. Computational screens of redox properties

1. Redox property screening using quantum mechanical methods

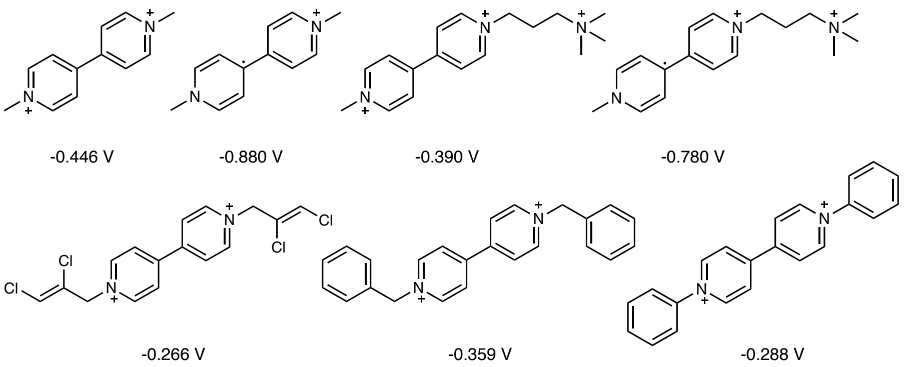

Supplementary Figure 4 | Viologens used for redox potential model development. Experimental redox potentials *vs.* SHE included below each molecule a previously reported.[12,13]

Starting with a general reaction scheme to describe single-electron redox processes (Eq. 1), redox potentials were obtained using the Nernst equation to obtain the Gibbs Free Energy change in solution (Eq. 2). Free energy values were calculated from geometry optimized structures based on density functional theory (DFT) carried out at the B3LYP[14-16] 6-31++G(d,p) level using an UltraFine integration grid, Grimme's third version of empirical correction for dispersion with Becke-Johnson damping (GD3BJ)[17], and implicit solvation model (SMD parametrization for water)[18] as implemented in Gaussian 09.[19] To simplify the DFT calculations, the 3-bromopropyl)trimethylammonium (TMAP) group of the synthesized compounds was replaced by a methyl group.

$$M^{n+} + e^{-} \rightarrow M^{(n-1)+} \quad (1)$$
$$E_{redox} = ((G_{ox} - G_{red}) / nF) - E_{SHE} \quad (2)$$

F = 23.06 kcal mol^{-1} V^{-1} is the Faraday constant. G_{ox} and G_{red}, given in kcal mol^{-1}, are the Gibbs free energies of both the oxidized and reduced states in solution, respectively. n = 1 is the number of electrons transferred. E_{SHE} = 4.43 V is the potential of the standard hydrogen electrode.[17]

Using a representative reference dataset of seven viologens for which a consistent set of experimental redox data was available (Supplementary Fig. 4), experimental redox potentials[12-13] were correlated with calculated redox potentials and a linear correlation (R^2 = 0.98) was obtained (Supplementary Fig. 5). Inspection of the linear expression revealed a gradient of 0.63 and an intercept of -0.024 (≈ 0). Given that, in an ideal case (*i.e.* $E_{exp} = E_{theo}$), these values would be 1 and 0 respectively, and that redox potentials are related to their corresponding free energies by Eq. 4, the deviation in the gradient obtained is indicative of differences in the calculated and experimental free energies (Eq. 5). That these differences can be captured by a constant dimensionless scaling factor of 0.63 suggest that simple corrections can be applied to obtain reliable estimates for experimental redox potential values based on computational input structures, despite the small double zeta basis set. Calculations performed at the ωB97X-D 6-311++G(3df,3pd) level for methyl viologen and its reduced forms yielded almost identical results to those obtained from the B3LYP 6-31++G(d,p) level, suggesting that more advanced computational approaches do not provide substantial improvements in accuracy.[1]

$$E_{exp} = 0.634E_{theo} - 0.024 \quad (3)$$
$$E = -\Delta G / nF \quad (4)$$
$$0.63 = \Delta G_{exp}/\Delta G_{theo} \quad (5)$$

Similar calculations performed using semiempirical tight binding (GFN2-xTB[21], GBSA implicit solvation for water) yielded weaker linear correlations ($R^2 = 0.85$) and much more pronounced deviations from ideality (Supplementary Fig. 5c,d) suggesting that such methods may not suitable for accurate redox potential screening calculations. As a result, DFT-based methods were used for all redox property calculations in the present work. Redox potentials were calculated using Eqs. 2 & 3 throughout.

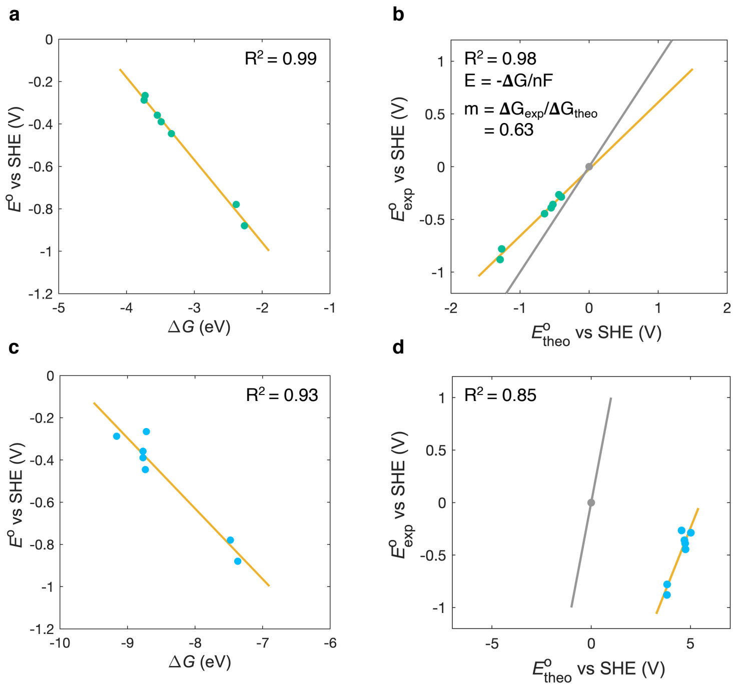

Supplementary Figure 5 | Correlations between theoretical and experimental viologen redox data. **(a)** Linear correlation between experimental redox potential and DFT-calculated free energy of reduction; **(b)** DFT-calculated redox potential. **(c)** Linear correlation between experimental redox potential and tight-binding-calculated free energy of reduction (**c**); **(d)** tight-binding-calculated redox potential. (0,0) (not fitted) and the linear expression for $E_{exp} = E_{theo}$ are included in grey.

2. Empirical correlations

To obtain predictive insights into the redox chemistry of viologens from which suitable candidates for library development can be selected, empirical methods suitable for high-throughput screening were developed. Leveraging reported experimental correlations between R-group Hammett constants and viologen redox potentials,[22] we generated a computational library of 48 viologens spanning five viologen subclasses (classical, ionic, aryl, *ortho* substituted and *meta* substituted) to determine whether such trends could be replicated *in silico*. These viologen subclasses, together with 'extended' viologens (see below) cover all viologen classes demonstrated for RFBs to date. Upon calculation of the respective redox potentials using DFT, and subsequent comparison with their corresponding R group *para* and *meta* Hammett constants from literature,[23] a series of linear correlations ($R^2 > 0.60$) were obtained for the first and second redox events for all viologen subclasses tested (Supplementary Fig. 6), consistent with experimental findings.[22] From these data, three general trends emerged.

First, as was previously observed,[22] it was found that negative Hammett constant values, indicative of electron donating character, shifted both first and second redox events to more negative potentials and *vice versa*, confirming that R-group substituents can be used to tune redox potentials over arbitrarily broad ranges. Given that Hammett constants for alkyl groups fall within a narrow range (–0.07 for methyl, –0.10 for *tert*-butyl; *meta* Hammett constant), this offers a possible reason why classical viologens used in RFBs to date have typically exhibit first electron redox potentials within a 0.11 V span (-0.35 V to -0.46 V).

Second, it was found that skeletal diversity in viologens (accounted for by analysis of *ortho* and *meta* substituted viologens), resulted in a negative shift in both first and second redox events over a similar Hammett constant range relative to classical viologen benchmarks. These findings are consistent with those obtained by Aziz and co-workers,[24] where introduction of *ortho* substituents to the bispyridinium core resulted in negative shifts in first and second redox potential relative to identical unsubstituted analogues.

Third, it was found that the gap between viologen redox events was largely independent of the R-group substituent used. The size of gap between redox events is known to influence both the complexity of the discharge profile, and the position of the comproportionation equilibrium between the three viologen oxidation states (section S6, Supplementary Information). As a result, an understanding of what factors narrow or widen this gap is critical for the selection of suitable candidate molecules for systematic investigations into viologen redox equilibria. For all viologen subclasses tested, no correlation between redox potential gap and Hammett constant was found ($R^2 < 0.4$ in all cases). Furthermore, no statistically significant difference (two-tailed difference of means t-tests; 95% significance level) in the gap between redox events was found for ionic viologens and aryl viologens relative to classical viologens (gaps of 0.37 ± 0.10 V, 0.36 ± 0.04 V, and 0.38 ± 0.08 V respectively). However, for *ortho* and *meta* substituted viologens, while no trend in redox potential gap was obtained as before, the absolute gap between redox events was found to be significantly higher (0.46 ± 0.04 V and 0.51 ± 0.21 V respectively). These results suggest that core skeletal diversity, rather than R group substitution, influences the redox potential gap in viologens (Supplementary Fig. 7).

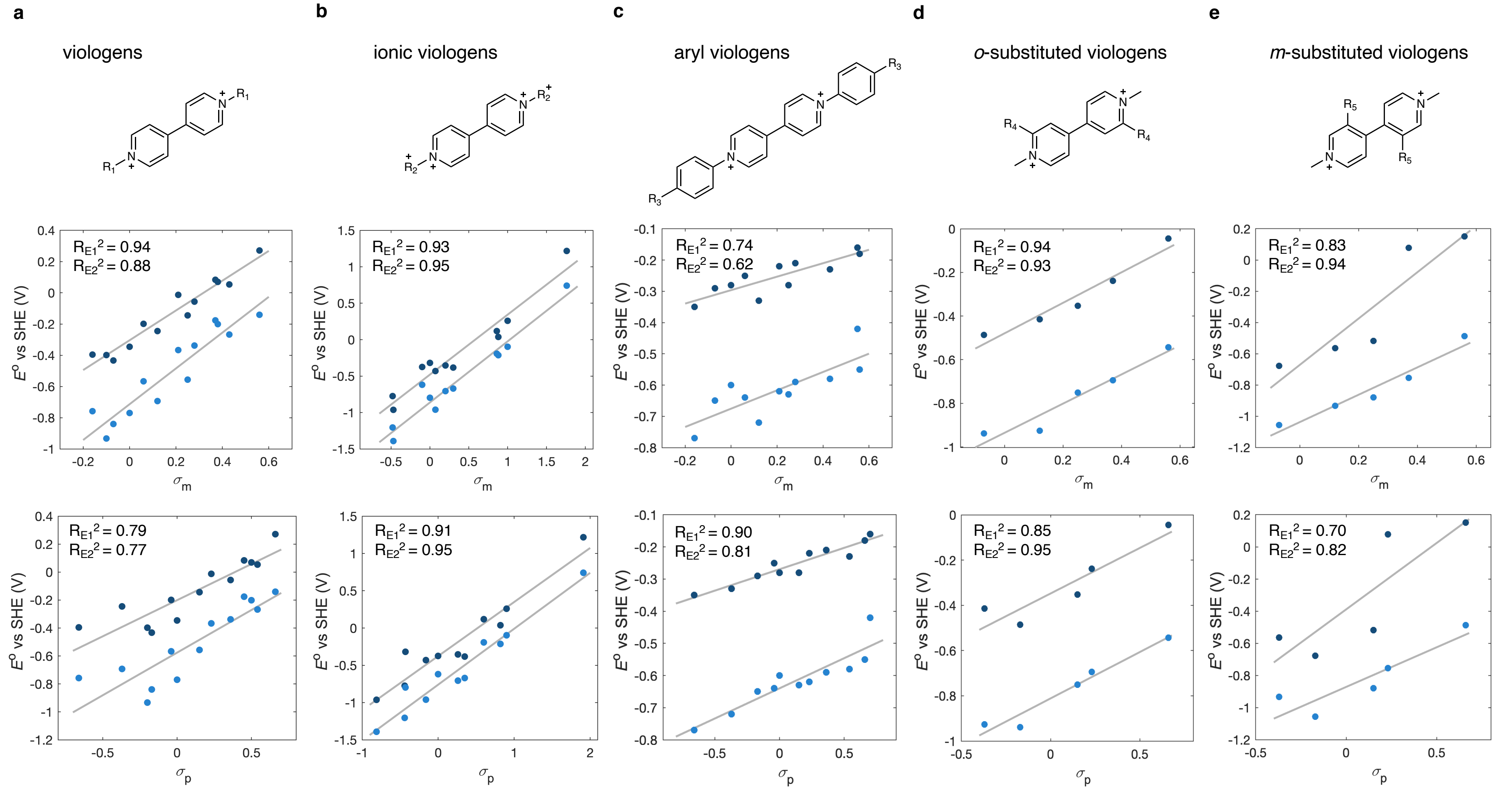

Supplementary Figure 6 | Semi-empirical models for viologen redox property estimation. Linear correlations obtained between DFT-calculated redox potential and *meta* and *para* Hammett constants (σ_m and σ_p respectively) for **(a)** viologens, **(b)** ionic viologens, **(c)** aryl viologens, **(d)** *ortho*-substituted viologens and **(e)** *meta*-substituted viologens. Dark blue circles indicate data corresponding to first reduction events. Light blue circles indicate data corresponding to second reduction events. Grey lines indicate least-squares linear fits to the respective data sets. R^2 values for fits corresponding to each redox event are indicated on the respective plots. R-groups used are given in Supplementary Table 1.

To determine the extent to which viologen core diversity could be used to tune, and especially narrow the gap between redox events, a further series of calculations was carried out on two representative classes of extended viologen known to possess narrow gaps between redox events: phenyl and thiophene-based ‘extended’ viologens. Using these two bispyridinium skeletal units, R-groups were systematically varied over a similar range to that used for classical viologens. As before, their *meta* Hammett constant values were compared to DFT-calculated redox potentials and good linear correlations were obtained throughout ($R^2 > 0.85$). In terms of redox potentials, similar trends with Hammett constant values were obtained indicating that the redox potentials of ‘extended’ viologens can also be tuned using R-group substituents. A negative shift in redox potential was also observed for ‘extended’ viologens relative to classical viologens, as was the case for *ortho* and *meta* substituted viologens. These findings are consistent with those from literature[10,11,25] and further indicate that similar enhancements in energy density afforded by substituted viologens are accessible by ‘extended’ analogues. However, for both classes of ‘extended’ viologen, redox potential gaps of 0.29 ± 0.05 V were obtained – significantly (95% significance level) lower than those obtained for classical viologens or any other viologen subclass tested. This suggests that the extended conjugation afforded by both homo- and heteroatom based aromatic cores can be used to narrow the gap between redox events. As no further correlations were obtained between redox potential gaps and R group Hammett constants, extended viologen potential gaps were found to be insensitive to R group substituents, confirming that core diversity rather than R-group substituents is responsible for these phenomena. Based on these findings, ‘extended’ viologens featuring aromatic cores were identified as promising candidates for subsequent investigation

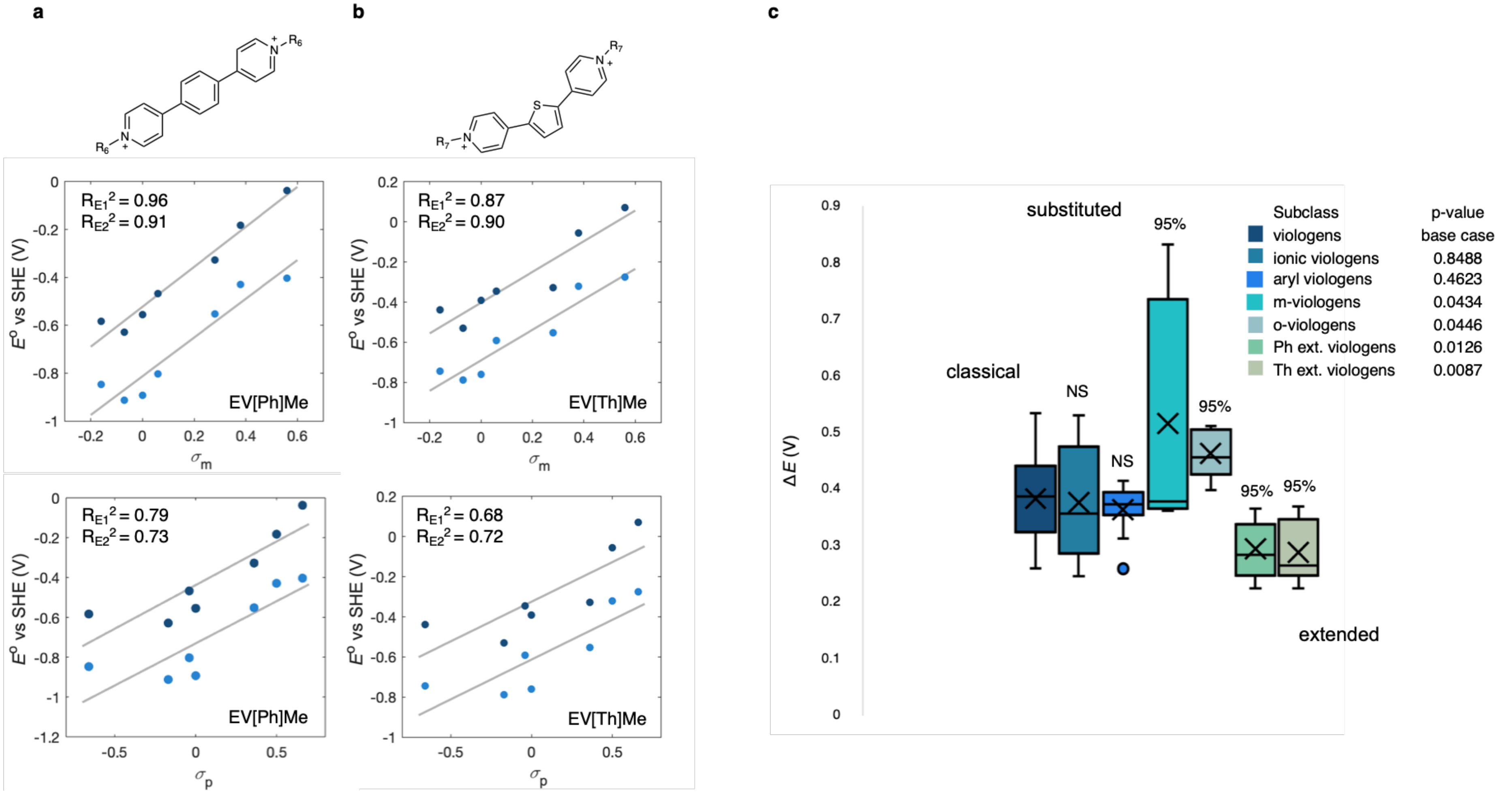

Supplementary Figure 7 | Semi-empirical models for extended viologen redox property estimation. Linear correlations obtained between DFT-calculated redox potential and *meta* and *para* Hammett constants (σ_m and σ_p respectively) for **(a)** phenyl, and **(b)** thiophene based compounds. **(c)** Box and whisker plots showing the gap between first and second redox events across viologens subclasses. Boxes indicate first, second and third quartiles. Crosses indicate arithmetic means. Whiskers indicate the minimum and maximum values within the dataset. Outliers are indicated as discrete datapoints. Two-tailed difference of means t-tests between viologens and various viologen subclasses revealed statistically significant differences (95% level) for substituted and extended viologens. Respective p-values for each subclass are shown

R-groups used in semi-empirical correlations

Supplementary Table 1 | R-groups and corresponding Hammett constant values used in semi-empirical correlations.

Viologens (R_1)	σ_M	σ_P	Ionic viologens (R_2)	σ_M	σ_P
H	0.00	0.00	O^-	-0.47	-0.81
Me	-0.07	-0.17	COO	-0.10	0.00
$CHCH_2$	0.06	-0.04	CH_2COO^-	0.07	-0.16
NH_2	-0.16	-0.66	$S(Me)_2^+$	1.00	0.90
OH	0.12	-0.37	PO_3H^-	0.20	0.26
SH	0.25	0.15	NNO_2^-	0.00	-0.43
CN	0.56	0.66	$B(OH)_3^-$	-0.48	-0.44
CF_3	0.43	0.54	NH_3^+	0.86	0.60
CCH	0.21	0.23	$N(Me)_3^+$	0.88	0.82
COMe	0.38	0.50	N_2^+	1.76	1.91
COOH	0.37	0.45	SO_3^-	0.30	0.35
$CONH_2$	0.28	0.36			
t-Bu	-0.10	-0.20			
Aryl viologens (R_3)	σ_M	σ_P	*o*-substituted viologens (R_4)	σ_M	σ_P
H	0.00	0.00	Me	-0.07	-0.17
Me	-0.07	-0.17	OH	0.12	-0.37
$CHCH_2$	0.06	-0.04	CN	0.56	0.66
NH_2	-0.16	-0.66	SH	0.25	0.15
OH	0.12	-0.37	CF_3	0.43	0.54
SH	0.25	0.15	Cl	0.37	0.23
CN	0.56	0.66	*m*-substituted viologens (R_5)	σ_M	σ_P
CF_3	0.43	0.54	Me	-0.07	-0.17
CCH	0.21	0.23	OH	0.12	-0.37
COF	0.55	0.7	CN	0.56	0.66
$CONH_2$	0.28	0.36	SH	0.25	0.15
			CF_3	0.43	0.54
			Cl	0.37	0.23

3. Singlet-triplet gap calculations

Singlet-triplet gaps (E_{ST}), were calculated by taking the difference in free energies obtained for optimized structures for both the singlet and triplet forms of the respective doubly reduced viologen species. As before, free energy values were calculated from geometry optimized structures based on DFT carried out at the UB3LYP/6-31++G(d,p) level using an UltraFine integration grid, GD3BJ and the SMD implicit solvation model as implemented in Gaussian 09.

4. NMR and EPR calculations

Gaussian 16 was used to calculate the NMR and EPR values of previously optimized structures (along with tetramethylsilane, TMS).[26] The same settings were used for NMR calculations as before, i.e. UB3LYP/6-31++G(d,p) level of theory with GD3BJ and SMD (water). For EPR calculations the EPR-III basis set was used instead of 6-31++G(d,p), except for sulfur atoms, where def2-TZVPP was used. In each of the EPR calculations the UB3LYP/6-31++G(d,p) optimized structure was used. The results are shown in Supplementary Tables 2-6.

Supplementary Table 2 | Calculated ^{1}H NMR shifts, given relative to TMS, of 10' (10 with Me instead of TMAP) in three oxidation states along with an inset of the structure and proton labels. Calculated isotropic Fermi contact couplings are reported in MHz, labelled with /EPR.

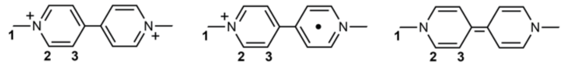

Number	**$10'^{2+}$** [ppm]	**$10'^{+\bullet}$** [ppm]	**10'** [ppm]	**$10'_{triplet}$** [ppm]	**$10'^{+\bullet}$**/EPR [MHz]	**$10'_{triplet}$** /EPR [MHz]
2	8.94	7.32	5.98	5.30	-3.92	-0.95
3	8.58	7.21	5.69	4.17	-4.49	-4.51
3	8.56	7.20	5.69	4.65	-4.55	-1.35
2	8.89	7.30	5.98	4.22	-3.87	-16.71
3	8.56	7.20	5.69	4.65	-4.55	-1.35
2	8.89	7.30	5.98	4.22	-3.87	-16.71
2	8.94	7.32	5.98	5.30	-3.92	-0.95
3	8.58	7.21	5.69	4.17	-4.49	-4.51
1	4.51	3.45	2.74	1.95	5.65	9.50
1	4.31	3.69	2.84	2.06	24.58	13.16
1	4.65	3.48	2.74	2.11	6.90	0.13
1	4.51	3.45	2.74	1.95	5.65	9.50
1	4.31	3.69	2.84	2.06	24.58	13.16
1	4.65	3.48	2.74	2.11	6.90	0.13

Supplementary Table 3 | Calculated ^{1}H NMR shifts, given relative to TMS, of 11' (11 with Me instead of TMAP) in three oxidation states along with an inset of the structure and proton labels. Calculated isotropic Fermi contact couplings are reported in MHz, labelled with /EPR.

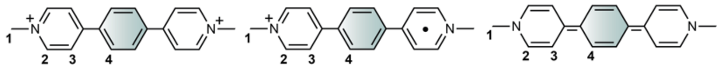

Number	**11'**$^{2+}$ [ppm]	**11'**$^{+\bullet}$ [ppm]	**11'** [ppm]	**11'**$_{triplet}$ [ppm]	**11'**$^{+\bullet}$ /EPR [MHz]	**11'**$_{triplet}$ /EPR [MHz]
2	8.75	7.44	6.35	5.82	-3.39	-7.23
3	8.53	7.59	6.36	6.34	-2.55	0.83
3	8.54	7.59	6.36	6.31	-2.63	0.81
2	8.74	7.41	6.35	5.82	-3.31	-7.05
4	8.46	7.76	6.61	7.21	-2.42	-2.19
4	8.46	7.76	6.61	7.15	-2.44	-2.21
4	8.46	7.76	6.61	7.21	-2.42	-2.19
4	8.46	7.76	6.61	7.15	-2.44	-2.21
3	8.53	7.59	6.36	6.34	-2.55	0.83
3	8.54	7.59	6.36	6.31	-2.63	0.81
2	8.75	7.44	6.35	5.82	-3.39	-7.23
2	8.75	7.41	6.35	5.82	-3.31	-7.06
1	4.32	3.56	2.95	2.66	5.04	3.13
1	4.30	3.51	2.95	2.64	3.69	3.22
1	4.54	3.77	3.11	2.79	16.86	15.41
1	4.30	3.51	2.95	2.64	3.70	3.22
1	4.32	3.56	2.95	2.66	5.04	3.13
1	4.54	3.77	3.11	2.79	16.86	15.42

Supplementary Table 4 | Calculated ^{1}H NMR shifts, given relative to TMS, of 13' (13 with Me instead of TMAP) in three oxidation states along with an inset of the structure and proton labels. Calculated isotropic Fermi contact couplings are reported in MHz, labelled with /EPR.

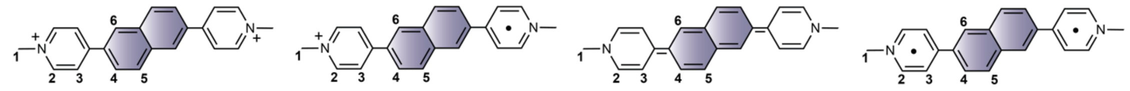

Number	**13'**$^{2+}$ [ppm]	**13'**$^{+•}$ [ppm]	**13'** [ppm]	**13'**$_{triplet}$ [ppm]	**13'**$^{+•}$ /EPR [MHz]	**13'**$_{triplet}$ /EPR [MHz]
2	8.73	7.55	6.58	6.01	-3.86	-7.10
3	8.60	7.66	6.54	6.60	-1.65	0.90
3	8.65	7.71	6.52	6.60	-2.37	0.11
2	8.71	7.54	6.61	6.14	-2.73	-5.45
4	8.54	7.84	6.81	7.45	0.24	-1.14
5	8.63	7.60	6.48	7.60	-2.42	-0.38
6	8.91	7.79	6.25	7.14	-5.49	-5.50
5	8.63	7.60	6.48	7.60	-2.42	-0.38
4	8.54	7.84	6.81	7.45	0.24	-1.14
6	8.91	7.79	6.25	7.14	-5.49	-5.50
3	8.65	7.71	6.52	6.60	-2.37	0.11
2	8.71	7.54	6.61	6.14	-2.73	-5.45
2	8.73	7.55	6.58	6.01	-3.86	-7.10
3	8.60	7.66	6.54	6.60	-1.65	0.90
1	4.33	3.62	3.06	2.81	4.21	3.65
1	4.24	3.59	3.06	2.78	3.44	2.82
1	4.52	3.83	3.25	2.95	14.77	14.73
1	4.24	3.59	3.06	2.78	3.44	2.82
1	4.52	3.83	3.25	2.95	14.77	14.73
1	4.33	3.62	3.06	2.81	4.21	3.65

Supplementary Table 5 | Calculated ^{1}H NMR shifts, given relative to TMS, of 17' (17 with Me instead of TMAP) in three oxidation states along with an inset of the structure and proton labels. Calculated isotropic Fermi contact couplings are reported in MHz, labelled with /EPR.

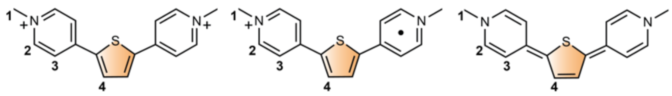

Number	**17'**$^{2+}$ [ppm]	**17'**$^{+\bullet}$ [ppm]	**17'** [ppm]	**17'**$_{triplet}$ [ppm]	**17'**$^{+\bullet}$ /EPR [MHz]	**17'**^{triplet}/EPR [MHz]
2	8.64	7.42	6.35	5.99	-3.46	-5.28
3	8.62	7.45	6.36	6.00	-2.51	0.00
3	8.11	6.76	5.43	5.87	-3.39	0.98
2	8.55	7.45	6.51	5.76	-2.29	-7.67
4	8.59	7.57	6.61	6.20	-2.57	-6.29
4	8.59	7.57	6.61	6.20	-2.57	-6.29
3	8.11	6.76	5.43	5.87	-3.39	0.98
3	8.62	7.45	6.36	6.00	-2.51	0.00
2	8.55	7.45	6.51	5.76	-2.29	-7.67
2	8.64	7.42	6.35	5.99	-3.46	-5.28
1	4.10	3.48	2.96	2.69	2.44	3.72
1	4.31	3.61	2.98	2.69	6.45	2.56
1	4.42	3.75	3.12	2.80	16.22	14.41
1	4.10	3.48	2.96	2.69	2.43	3.72
1	4.31	3.61	2.98	2.69	6.45	2.56
1	4.42	3.75	3.12	2.80	16.22	14.41

Supplementary Table 6 | Calculated ^{1}H NMR shifts, given relative to TMS, of 18' (18 with Me instead of TMAP) in three oxidation states along with an inset of the structure and proton labels. Calculated isotropic Fermi contact couplings are reported in MHz, labelled with /EPR.

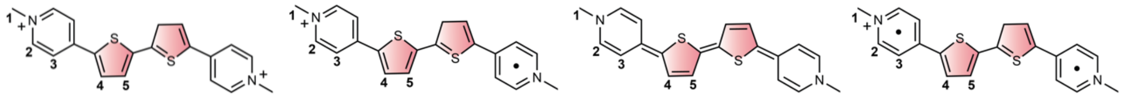

Number	**18'**$^{2+}$ [ppm]	**18'**$^{+\bullet}$ [ppm]	**18'** [ppm]	**18'**$_{triplet}$ [ppm]	**18'**$^{+\bullet}$ /EPR [MHz]	**18'**$_{triplet}$ /EPR [MHz]
2	8.48	7.51	6.65	6.33	-2.77	-4.42
3	8.45	7.51	6.65	6.37	-1.92	-0.15
3	7.95	6.86	5.75	6.06	-2.48	0.23
2	8.46	7.56	6.78	6.28	-1.86	-5.09
4	8.39	7.65	6.95	6.36	-1.81	-7.59
5	7.91	7.10	6.25	6.79	-1.60	-0.18
1	4.14	3.60	3.12	2.93	3.20	3.21
1	4.13	3.60	3.13	2.92	3.37	2.68
1	4.37	3.83	3.33	3.10	12.67	12.40
5	7.91	7.10	6.25	6.79	-1.61	-0.18
4	8.39	7.65	6.95	6.36	-1.81	-7.59
3	7.95	6.86	5.75	6.06	-2.48	0.23
3	8.45	7.51	6.65	6.37	-1.92	-0.15
2	8.46	7.56	6.78	6.28	-1.86	-5.09
2	8.48	7.51	6.65	6.33	-2.77	-4.42
1	4.14	3.60	3.12	2.93	3.20	3.21
1	4.13	3.60	3.13	2.92	3.37	2.68
1	4.37	3.83	3.33	3.10	12.67	12.40

S5. Radical concentration calculations

These are described in detail elsewhere.[27,28] Briefly, radical concentrations (C_s) were quantified by taking the double integral (DI) of the EPR signal according to:

$$DI = C_{ns}\frac{n_B Q B_m \sqrt{P} S(S+1) C_s v}{f(B_1, B_m)}$$

where C_{ns} is a constant including the normalized spectrometer settings, *i.e.* sweep time and number of accumulations, P is the microwave power, B_m is the modulation amplitude, Q is the resonator's quality factor, n_B is the Boltzmann factor for temperature dependence, S is the total electron spin, v is the volume of the sample, and $f(B_1,B_m)$ is the spatial distribution of microwave and modulation field in the sample.

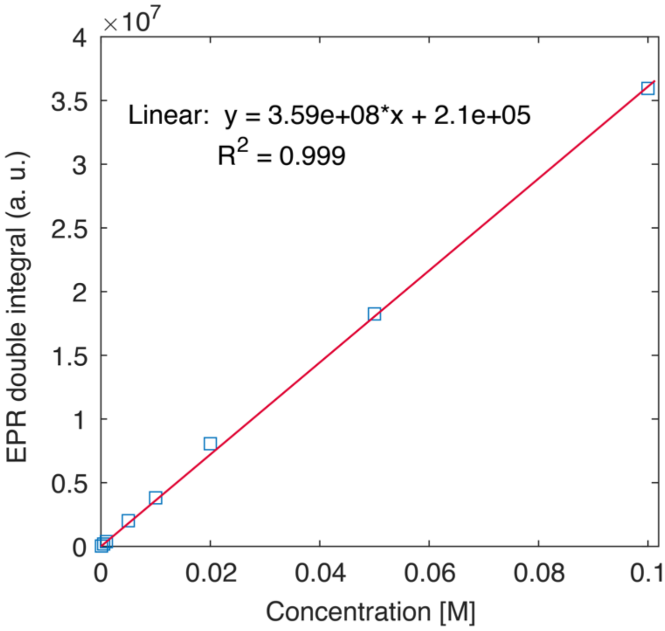

Supplementary Figure 8 | EPR calibration curve based on 4-hydroxy TEMPO standard solutions. The double integral of the first derivative spectra as a function of the 4-OH-TEMPO concentration. The yellow line represents the result of a least-squares fit, with a slope of 3.59×10^5 mM^{-1} and an R^2 of 0.999.

S6. Equilibrium models and fits to experimental data

These are described in detail elsewhere.[27,28] Briefly, the difference between redox events is related to the comproportionation equilibrium constant K_c by:

$$E_1 - E_2 = \frac{RT\ln(K_c)}{F}$$

For the viologen comproportionation equilibrium:[28]

$$V^{4+} + V^{2+} \rightleftharpoons 2V^{3+\bullet}, K_c = \frac{[V^{3+\bullet}]^2}{[V^{2+}][V^{4+}]}$$

fits to experimental radical concentration data were obtained by:

$K_c \neq 4$

$$x = \frac{K_c \pm \sqrt{{K_c}^2 - (K_c - 4)(2n - n^2)K_c}}{K_c - 4}$$

$K_c = 4$

$$x = n - \frac{1}{2}n^2$$

Where n is the number of electrons that have been removed from the system, starting from 100% SOC and x is the fraction of radicals.

For π-dimerization equilibria, K_d values were estimated as follows. At 50% SOC, 1 electron equivalent of the starting concentration of viologen, V_0, has been added to create reduced species, thus giving the previous result[28]:

$$2K_d[V^{3+\bullet}]^2 + [V^{3+\bullet}] - 2\frac{[V^{3+\bullet}]}{\sqrt{K_c}} - [V_0] = 0$$

As the initial concentration (V_0), K_c and the radical concentration ($V^{3+\bullet}$) at this state of charge is known, the dimerisation constant can be calculated with:

$$K_d = \frac{\left([V_0] + 2\frac{[V^{3+\bullet}]}{\sqrt{K_c}} - [V^{3+\bullet}]\right)}{2[V^{3+\bullet}]^2}$$

Extracting the K_c value from the radical concentration for **10**, **11** and **13**, gives an average K_c of 0.7, 0.2 and 0.002, respectively. The notably low values of **13** are also unreliable, as there is a large asymmetry in the radical concentration as a function of SOC (Supplementary Figs. 9-11) meaning that some assumptions are likely being violated.

The fitting of the cyclic voltammetric data was done in a similar manner as reported previously.[27] A quasi-reversible $1e^- + 1e^-$ reaction with a fixed α along with a single diffusion coefficient for all three species was chosen as the equation governing the fits. This was done for all 10 CV cycles. Supplementary Fig. 12 shows an example of such a fit for the first cycle of **10**.

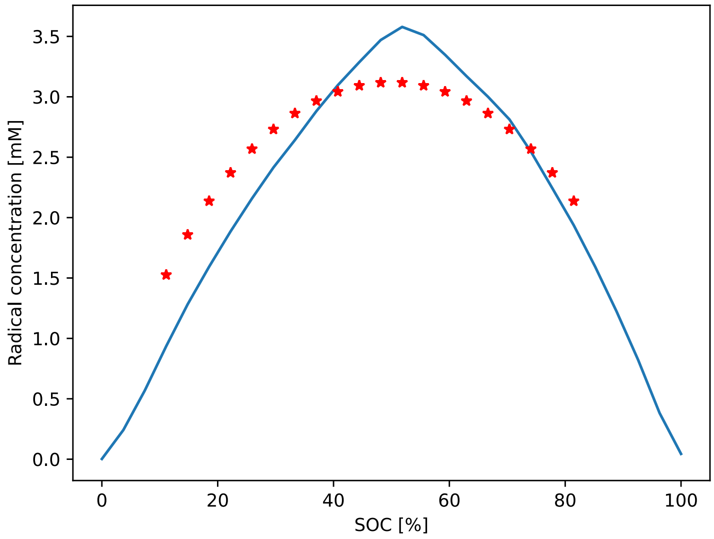

Supplementary Figure 9 | Radical concentration for the first charge cycle as a function of SOC for 10. The experimental data is blue, while fitted curve is shown in red stars.

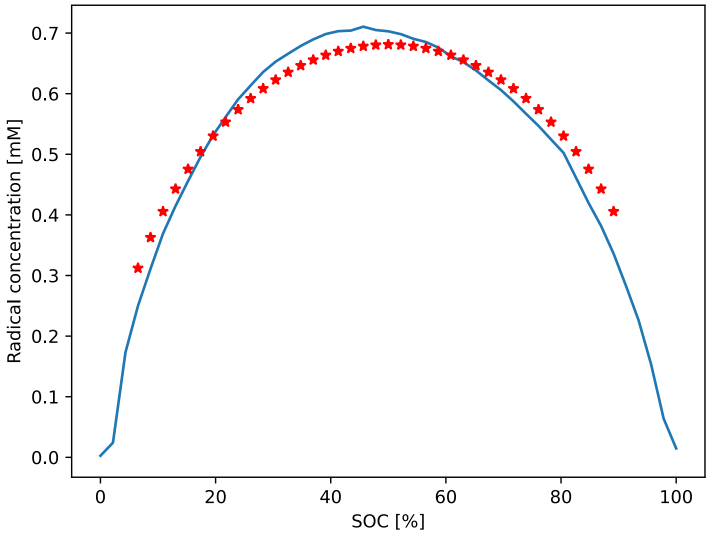

Supplementary Figure 10 | Radical concentration for the first charge cycle as a function of SOC for 11. The experimental data is blue, while fitted curve is shown in red stars.

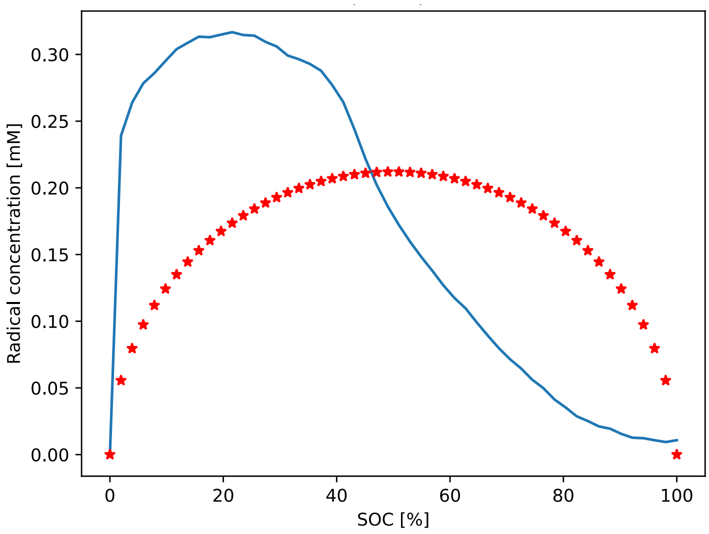

Supplementary Figure 11 | Radical concentration for the first charge cycle as a function of SOC for 13. The experimental data is blue, while fitted curve is shown in red stars.

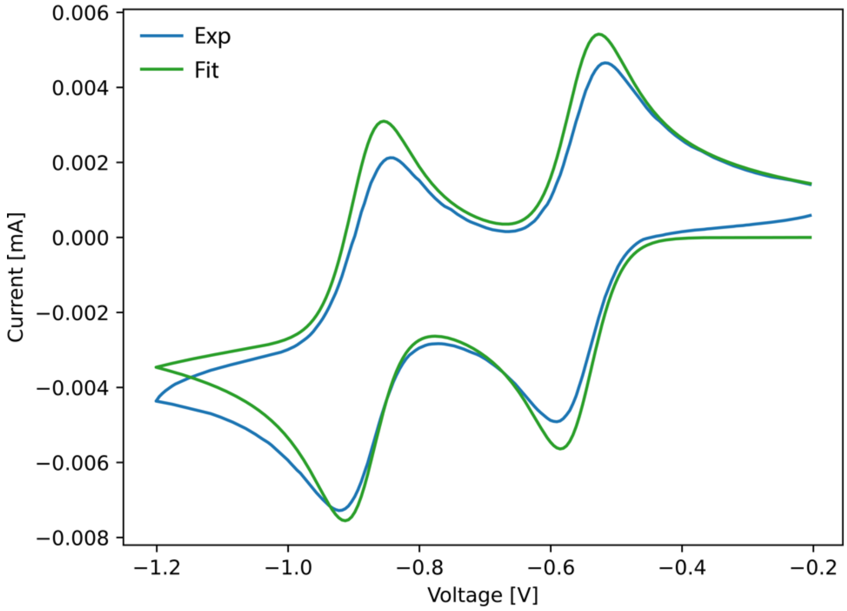

Supplementary Figure 12 | A CV curve and its fitted counterpart for a cycle of 10. Experimental data are plotted in blue, while that data fit is plotted in green.

S7. Evidence for other associated structures

In all cases *operando* experiments provided at least partial evidence to support the existence of additional associated structures (beyond the π-dimers). This included H-D exchange at the preferred position for σ-dimerization previously observed in methyl viologen[30] (Supplementary Fig. 13) and the decreasing diffusivity of the viologen ions from 50 – 100 % SOC for **10** and **11** (Extended Data Table 1). We note that higher order associated structures (trimers, tetramers, oligomers, *etc.*), while expected to be suppressed through inclusion of TMAP groups,[31] cannot be ruled out either.[30,31] However, decreasing diffusivity of **11** as the SOC is increased from 50 – 100 % suggests that association of doubly reduced species is likely to be more pronounced than association of corresponding singly reduced species.

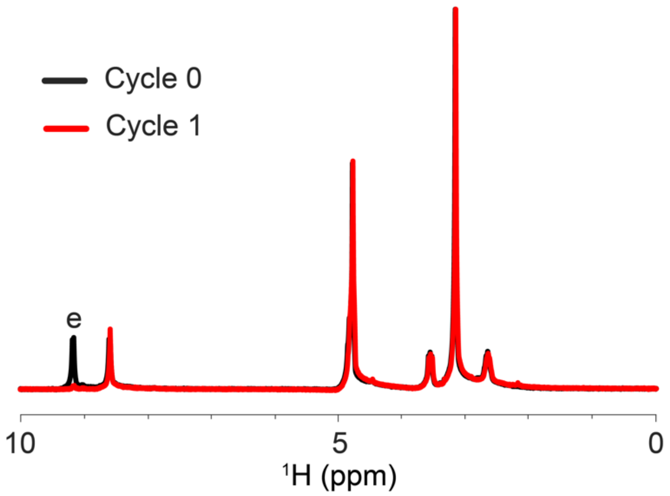

Supplementary Figure 13 | Overlaid 1D ^{1}H NMR spectra showing near complete H-D exchange for proton e of 10 during the first charge-discharge cycle.

S8. Thermodynamics of dimerization and stability to dioxygen

Density Functional Theory (DFT) thermodynamic analysis

To find the optimized dimer structure, an initial potential energy surface scan to explore the angle and distance between two modified **11** molecules (where the TMAP moieties are replaced with CH_3 groups; denoted further as **11'**) were performed. The electronic states explored were:

(i) $(\mathbf{11'})_2^{4+}$ (a dimer formed from two unreduced **11'** molecules),
(ii) $^{1}(\mathbf{11'})_2^{2+}$ (a dimer, in a singlet state, stemming from either two singly reduced **11'** species or one doubly reduced and one unreduced **11'** species),
(iii) $^{3}(\mathbf{11'})_2^{2+}$ (a dimer, in a triplet state, stemming from either two singly reduced **11'** species or one doubly reduced and one unreduced **11'** species),
(iv) $^{1}(\mathbf{11'})_2^{0}$ (a dimer, in a singlet state, stemming from two doubly reduced **11'** species),
(v) $^{3}(\mathbf{11'})_2^{0}$ (a dimer, in a triplet state, stemming from two doubly reduced **11'** species).

Single point calculations were performed for all the selected combinations of angles (0°, 15°, 30°, 45°, 60°, 75°, 90°) and distances (2.5, 2.75, 3.0, 3.25, 3.5, 3.75, 4.0, 4.25, 4.5, 4.75, 5.0, 6.0 Å). The Orca program package, version 5.0.3, was used for all of this section.[32] The level of theory was B3LYP/def2-TZVP with the D3BJ dispersion correction.[17,33] The default RIJCOSX and grid settings were used. The SMD implicit solvation model for water was also used.[18]

As shown in Supplementary Fig. 14 and summarized in Supplementary Table 7, these scans showed slight variations in what structures would be suitable initial guesses for geometry optimizations for the π-dimers. The selected structures were then optimized and confirmed to be minima *via* their frequencies.

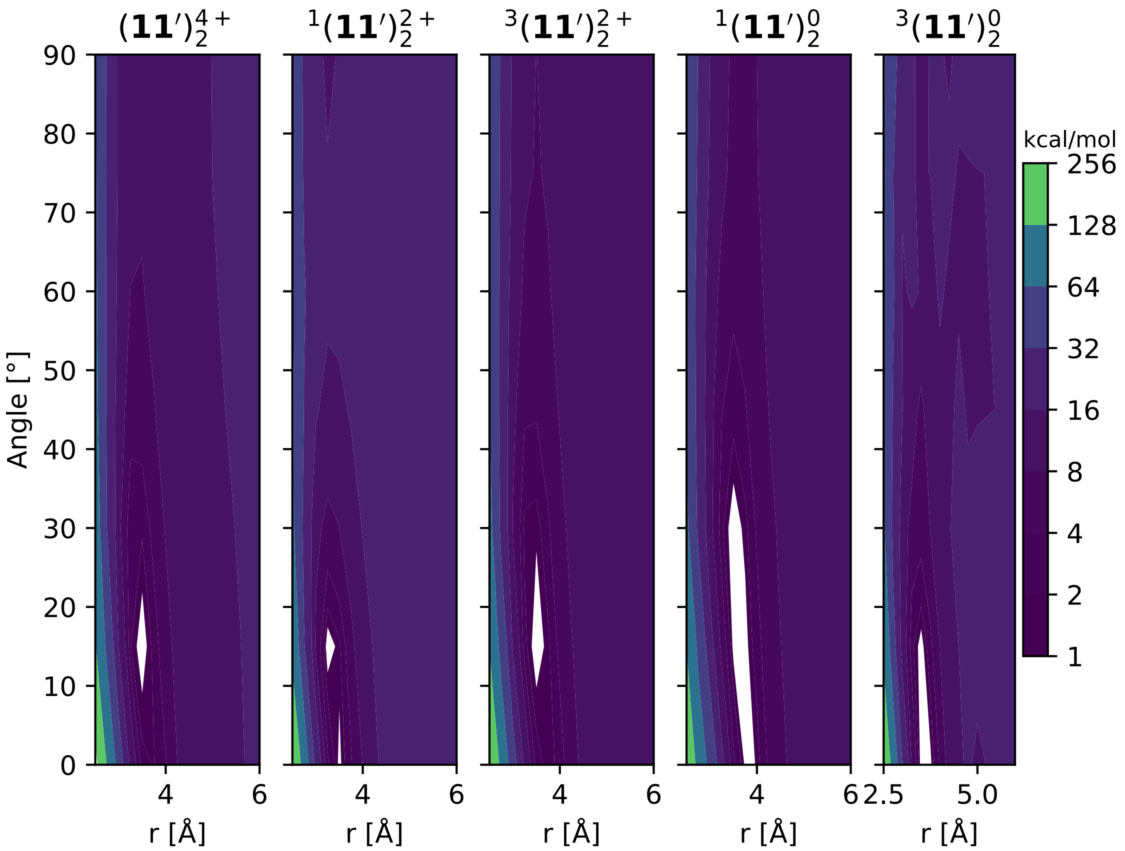

Supplementary Figure 14 | The potential energy surface for the various angles and distances that were used for the various electronic states of the dimers of 11', i.e. (11')$_2^{4+}$, ^{1}(11')$_2^{2+}$, ^{3}(11')$_2^{2+}$, ^{1}(11')$_2^0$ and ^{3}(11')$_2^0$. In each case, there is a shallow minimum (in white) which is used as a starting point for further geometry optimizations.

Supplementary Table 7 | The combination of angle and distance that gave the lowest energy for each of the electronic states explored for the dimer of 11' (a variant of 11 where Me replaces the TMAP moieties).

State	Angle [°]	Distance [Å]	E [a.u.]
(**11'**)$_2^{4+}$	15	3.5	-1611.69676551
^{1}(**11'**)$_2^{2+}$	15	3.25	-1611.96403862
^{3}(**11'**)$_2^{2+}$	15	3.5	-1611.95588393
^{1}(**11'**)$_2^0$	30	3.5	-1612.15712603
^{3}(**11'**)$_2^0$	0	3.5	-1612.14032339

The calculated singlet-triplet gap (ΔE_{ST}) of the dimers (Supplementary Table 8) is relatively small, as it is -2.90 and -12.03 kcal mol^{-1} for the (**11'**)$_2^{2+}$ and (**11'**)$_2^0$ systems, respectively. The value for (**11'**)$_2^0$ is very close to that of the monomer (-12.3 kcal mol^{-1}; Fig. 1e), which is also a fully reduced system.

In each case, the dimerization energy is negative (Supplementary Table 9) showing that there is a general propensity in the system for dimerization. It must be noted that there is a large difference in the electronic (ΔE) and Gibbs free (ΔG) energies, with the magnitude of the latter

being far lower in each case. In most cases, the calculated Gibbs free energy is relatively low, and considering thermal effects, there would not be appreciable amounts of most of the dimer types in solution.

We note, however, that at intermediate SOCs dimers involving mixed species are favored, as both $^{1}(\mathbf{11'})_2^{2+}$ and $^{3}(\mathbf{11'})_2^{2+}$ have negative Gibbs free energies, -15.30 and -25.43 kcal mol^{-1}, respectively.

Supplementary Table 8 | The singlet-triplet gap ($\Delta E_{ST} = E_{singlet} - E_{triplet}$), given for electronic, ΔE_{ST}, and Gibbs free energies, ΔG_{ST}, of the π-dimer systems, $^{1}(11')_2^{2+}$–$^{3}(11')_2^{2+}$ and $^{1}(11)_2^{0}$–$^{3}(11')_2^{0}$.

Charge	ΔE_{ST} [kcal mol^{-1}]	ΔG_{ST} [kcal mol^{-1}]
+2	-4.32	-2.90
0	-13.73	-12.03

Supplementary Table 9 | Electronic and Gibbs free energies of dimerization, for the various electronic states of 11'. Here, the table shows the A+B → AB reaction, with values calculated as $E_{dim}=E_{AB}-E_A-E_B$

A	B	AB	ΔE [kcal mol^{-1}]	ΔG [kcal mol^{-1}]
$\mathbf{11'}^{2+}$	$\mathbf{11'}^{2+}$	$(\mathbf{11'})_2^{4+}$	-21.17	-3.76
$\mathbf{11'}^{2+}$	$^{1}\mathbf{11'}^{0}$	$^{1}(\mathbf{11'})_2^{2+}$	-34.93	-15.30
$\mathbf{11'}^{2+}$	$^{3}\mathbf{11'}^{0}$	$^{3}(\mathbf{11'})_2^{2+}$	-45.90	-25.43
$^{1}\mathbf{11'}^{0}$	$^{1}\mathbf{11'}^{0}$	$^{1}(\mathbf{11'})_2^{0}$	-19.42	-1.11
$^{1}\mathbf{11'}^{0}$	$^{3}\mathbf{11'}^{0}$	$^{3}(\mathbf{11'})_2^{0}$	-20.98	-2.11
$\mathbf{11'}^{1+\bullet}$	$\mathbf{11'}^{1+\bullet}$	$^{1}(\mathbf{11'})_2^{2+}$	-25.68	-4.50
$\mathbf{11'}^{1+\bullet}$	$\mathbf{11'}^{1+\bullet}$	$^{3}(\mathbf{11'})_2^{2+}$	-21.36	-1.60

To study the origin of the O_2 stability of **11**, the electron affinities (the difference in Gibbs free energy between reduced and oxidized forms) of the involved species were calculated. Supplementary Table 10 shows the results for the various **11'** species, while for oxygen, $^{3}O_2$ to $O_2^{-\bullet}$, the electron affinity is -85.85 kcal mol^{-1} (calculated at the same level of theory). The calculated oxidative power of oxygen per mole of dioxygen is thus:

(i) for π-dimer species:
$2O_2 + {}^{1}(\mathbf{11'})_2^{2+} \rightarrow 2O_2^{-\bullet} + (\mathbf{11'})_2^{4+}$
$\Delta G/2 = [2*(-85.85) + 159.35]/2 = -6.18$ kcal mol^{-1}

(ii) for monomeric radical species:
$O_2 + \mathbf{11'}^{1+\bullet} \rightarrow O_2^{-\bullet} + \mathbf{11'}^{2+}$
$\Delta G = (-85.85) + 79.30 = -6.55$ kcal mol^{-1}

On the basis of the above DFT thermodynamic analysis, a small driving force for oxygen reduction was obtained. However, for the dimer, as this reaction would require two O_2 with each dimer, it is likely that the kinetics will be slower than in the monoradical case. Additionally, DFT does not fully account for electron correlation effects, which may have an effect here (see *CCSD(T) calculations using the Local Energy Decomposition analysis* below).

Supplementary Table 10 | The free energies of electron affinity/reduction of 11' monomers (11' is a modified version of 11 where CH_3 replaces the TMAP moieties) and the various dimers of 11'. While the singlet states of the dimers are energetically favored (in italics), the triplet states are given here for completeness.

Monomer electron affinity		ΔG_{EA} [kcal mol^{-1}]
$\mathbf{11'}^{2+}\rightarrow\mathbf{11'}^{+\bullet}$		-79.30
$\mathbf{11'}^{+\bullet}\rightarrow{}^{1}\mathbf{11'}^{0}$		-68.50
$\mathbf{11'}^{+\bullet}\rightarrow{}^{3}\mathbf{11'}^{0}$		-55.48
Dimer electron affinity	ΔG_{EA} [kcal mol^{-1}]	$\Delta G_{EA}/2$ [kcal mol^{-1}]
$(\mathbf{11'})_2^{4+}\rightarrow{}^{1}(\mathbf{11'})_2^{2+}$	*-159.35*	*-79.67*
$(\mathbf{11'})_2^{4+}\rightarrow{}^{3}(\mathbf{11'})_2^{2+}$	-156.45	-78.22
${}^{1}(\mathbf{11'})_2^{2+}\rightarrow{}^{1}(\mathbf{11'})_2^{0}$	*-133.61*	*-66.81*
${}^{1}(\mathbf{11'})_2^{2+}\rightarrow{}^{3}(\mathbf{11'})_2^{0}$	-121.58	-60.79
${}^{3}(\mathbf{11'})_2^{2+}\rightarrow{}^{1}(\mathbf{11'})_2^{0}$	-136.51	-68.26
${}^{3}(\mathbf{11'})_2^{2+}\rightarrow{}^{3}(\mathbf{11'})_2^{0}$	-124.48	-62.24

CCSD(T) calculations using the Local Energy Decomposition method

To further analyze the energetics of the dimers, the Local Energy Decomposition method (LED) was used,[34] as implemented in Orca. The previously DFT optimized geometries of the singlet dimers were used for this analysis. LED uses a very high level of theory, DLPNO-CCSD(T)[35] to separate terms that contribute to the electronic energy. The cc-pVDZ basis set (the smaller basis set was chosen due to the high computational expense of the coupled cluster method) was used along with the cc-pVDZ/C complement and cc-pVTZ/JK fitting basis set for the RIJK steps. The VeryTightSCF convergence criteria was used along with the TightPNO option. The implicit SMD solvation model of water was also used.

Supplementary Table 11 | The correlation energy terms that were extracted from LED calculations for the $(11')_2^{4+}$, ${}^{1}(11')_2^{2+}$ and ${}^{1}(11')_2^{0}$ systems. All energies are in atomic units.

System	Charge transfer 1 to 2	Charge transfer 2 to 1	Dispersion 1,2	Intra fragment 1	Intra fragment 2
$(\mathbf{11'})_2^{4+}$	-0.005382906	-0.006151134	-0.03069283	-2.751646984	-2.751019298
${}^{1}(\mathbf{11'})_2^{2+}$	-0.14353004	-0.027584528	-0.038605259	-2.726243825	-2.674213732
${}^{1}(\mathbf{11'})_2^{0}$	-0.006005	-0.005778662	-0.029310095	-2.802877093	-2.803083922

The key difference between the systems is the high charge transfer energy term of the ${}^{1}(\mathbf{11'})_2^{2+}$ π-dimer system (Supplementary Table 11). The charge transfer term corresponds to a dynamical charge polarisation,[36] thus the dimerization can be thought of as a charge transfer process where the stabilization is partially driven by such electron correlation effects (Supplementary Figs. 15-17). Though it must be noted, as per Supplementary Table 9, the ΔG is quite different from the ΔE, showing that some thermodynamic effects must be taken into account to get the full image. Notwithstanding, such electron correlation effects, which serve to significantly lower the overall energy of the π-dimers, may result in their reduced reactivity towards dioxygen.

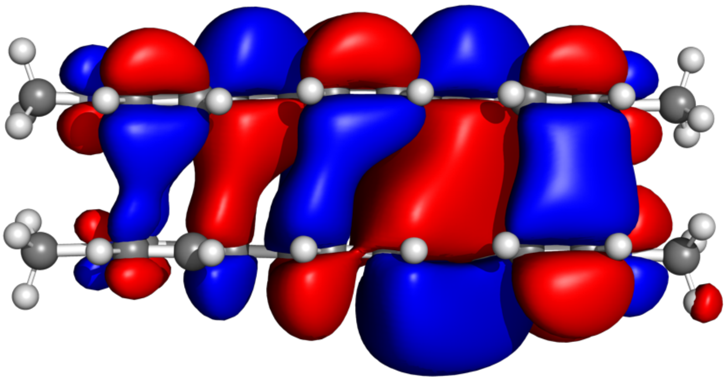

Supplementary Figure 15 | The CCSD(T)-calculated HOMO of $^{1}(\mathbf{11'})_2^{2+}$.

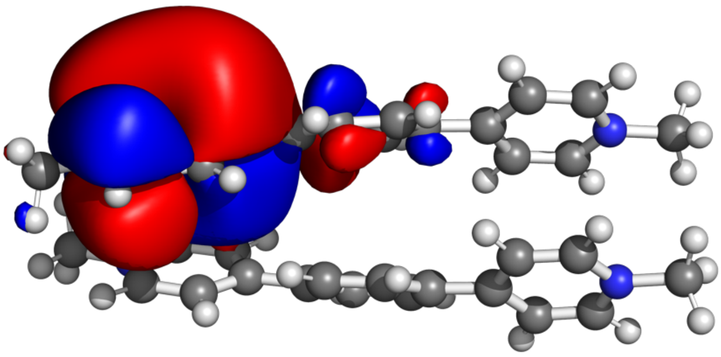

Supplementary Figure 16 | The CCSD(T)-calculated HOMO of $(\mathbf{11'})_2^{4+}$.

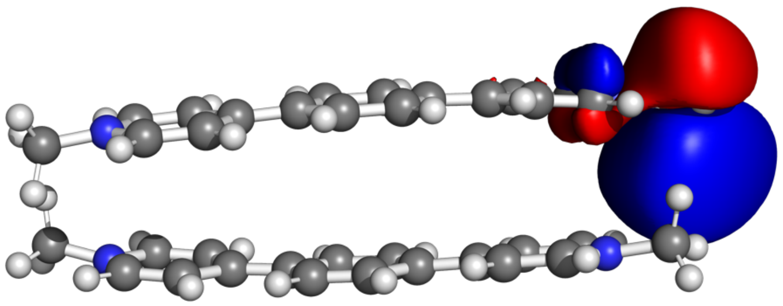

Supplementary Figure 17 | The CCSD(T)-calculated HOMO of $^{1}(\mathbf{11'})_2^{0}$.

S9. References

1. Sokołowski, K., *et al.* Nanoparticle surfactants for kinetically arrested photoactive assemblies to track light-induced electron transfer. *Nat. Nanotechnol.* 16, 1121-1129 (2021).
2. Wu, G., Olesińska, M., Wu, Y., Matak-Vinkovic, D., & Scherman, O.A. Mining 2:2 Complexes from 1:1 Stoichiometry: Formation of Cucurbit[8]uril-Diarylviologen Quaternary Complexes Favored by Electron-Donating Substituents. *J. Am. Chem. Soc.* 139, 3202-3208 (2017)
3. Olesińska, M., *et al.* Modular Supramolecular Dimerization of Optically Tunable Extended Aryl Viologens. *Chem. Sci.*, 10, 8806-8811 (2019)
4. Olesińska, M. *Design, Synthesis and Characterisation of π-Extended Viologen-Based Molecular and Supramolecular Complexes with Cucurbit[n]urils,* PhD Thesis, University of Cambridge, Cambridge UK (2019) https://doi.org/10.17863/CAM.39981.
5. Wu, G., *et al.* Cucurbit[8]uril-Mediated Pseudo [2,3] Rotaxanes. *Chem. Commun.* 55, 13227-13230 (2019)
6. Graf, A. M. *Development of Extended Viologens Towards Applications in Aqueous Organic Redox Flow Batteries*, Master's thesis, University of Cambridge, Cambridge UK (2019).
7. Wu, G. *et al.* Controlling the Structure and Photophysics of Fluorophore Dimers Using Multiple Cucurbit[8]uril Clampings. *Chem. Sci.* 11, 812-825 (2020)
8. Wu, G., Huang, Z., & Scherman, O. A. Quantitative Supramolecular Heterodimerization for Efficient Energy Transfer. *Angew. Chem. Int. Ed.* 59, 15963-15967 (2020)
9. Beh, E. S. et al. A neutral pH aqueous organic–organometallic redox flow battery with extremely high capacity retention. ACS Energy Lett. 2, 639–644 (2017).
10. Hu, S., *et al.* Phenylene-Bridged Bispyridinium with High Capacity and Stability for Aqueous Flow Batteries. *Adv. Mater.* 33, 2005839 (2021).
11. Huang, M., *et al.* Five-Membered-Heterocycle Bridged Viologen with High Voltage and Superior Stability for Flow Battery. *Adv. Funct. Mater.* 32, 2111744 (2022).
12. DeBruler, C., *et al.* Designer two-electron storage viologen anolyte materials for neutral aqueous organic redox flow batteries *Chem* 3, 961-978 (2017).
13. Bird, C. L., & Kuhn, A. T., Electrochemistry of the viologens *Chem. Soc. Rev.* 10, 49-82 (1981).
14. Becke, A. Density-functional thermochemistry. III. The role of exact exchange *J. Chem. Phys.* 98, 5648 (1993).
15. Stephens, P.J., *et al.* Ab initio calculation of vibrational absorption and circular dichroism spectra using density functional force fields *J. Phys. Chem.* 98, 11623-11627 (1994).
16. Kim, K., & Jordan, K. D. Comparison of density functional and MP2 calculations on the water monomer and dimer. *J. Phys.Chem.* 98, 10089-10094 (1994).
17. Grimme, S., Ehrlich, S., & Goerigk, L. Effect of the damping function in dispersion corrected density functional theory *J. Comput. Chem.* 32, 1456-65 (2011).
18. Marenich, A.V., Cramer, C.J., & Truhlar, D.G. Universal solvation model based on solute electron density and on a continuum model of the solvent defined by the bulk dielectric constant and atomic surface tensions *J. Phys. Chem. B* 113, 6378-6396 (2009).
19. Gaussian 09, Revision E.01, Frisch, M. J., *et al.* Gaussian, Inc., Wallingford CT, 2013.
20. Reiss, H., & Heller, A. The absolute potential of the standard hydrogen electrode: a new estimate. *J. Phys. Chem.* 89, 4207-4213 (1985).
21. Bannwarth, C., Ehlert, S., & Grimme, S. GFN2-xTB—An accurate and broadly parametrized self-consistent tight-binding quantum chemical method with multipole electrostatics and density-dependent dispersion contributions. *J. Chem. Theory Comput.* 15, 1652-1671 (2019).
22. Hünig, S., & Schenk, W. Über zweistufige Redoxsysteme, XXVI. Einfluß von N-Substituenten in 4,4′-Bipyridylen auf das Redoxverhalten, die Radikalstabilität und die Elektronenspektren. *Liebigs Ann. Chem.* 1979, 1523-1533 (1979).
23. Hansch, C., Leo, A., & Taft, R. W. A survey of Hammett substituent constants and resonance and field parameters. *Chem. Rev.* 91, 165-195 (1991).
24. Jin, S., *et al.* Near neutral pH redox flow battery with low permeability and long-lifetime phosphonated viologen active species. *Adv. Energy Mater,* 10, 2000100 (2020).
25. Luo, J., *et al.* A π-conjugation extended viologen as a two-electron storage anolyte for total organic aqueous redox flow batteries. *Angew. Chem. Int. Ed.*57, 231-235 (2018).
26. Gaussian 16, Revision C.01, Frisch, M. J., *et al.* Gaussian, Inc., Wallingford CT, 2016.

27. Zhao, E. W., *et al.* In situ NMR metrology reveals reaction mechanisms in redox flow batteries. *Nature* 579, 224-228 (2020).
28. Zhao, E. W., *et al.* Coupled in situ NMR and EPR studies reveal the electron transfer rate and electrolyte decomposition in redox flow batteries. *J. Am. Chem. Soc.* 143, 1885-1895 (2021).
29. Monk, P. M. S., *et al.* Evidence for the Product of the Viologen Comproportionation Reaction Being a Spin-Paired Radical Cation Dimer. *J. Chem. Soc. Perkin Trans.* 2, 2039-2041 (1992).
30. Xiang, Z., *et al*. Aggregation of Electrochemically Active Conjugated Organic Molecules and Its Impact on Aqueous Organic Redox Flow Batteries. *Angew. Chem. Int. Ed.* 62, e202214601 (2023).
31. Nolte, O., *et al*. State of Charge and State of Health Assessment of Viologens in Aqueous-Organic Redox-Flow Electrolytes Using In Situ IR Spectroscopy and Multivariate Curve Resolution. *Adv. Sci.* 9, 2200535 (2022).
32. Neese, F. Software update: The ORCA program system—Version 5.0. *Wiley Interdiscip. Rev. Comput. Mol. Sci.*12, e1606 (2022).
33. Grimme, S., *et al.* A consistent and accurate *ab initio* parametrization of density functional dispersion correction (DFT-D) for the 94 elements H-Pu. *J. Chem. Phys.* 132, 154104 (2010).
34. Schneider, W. B., *et al.* Decomposition of Intermolecular Interaction Energies within the Local Pair Natural Orbital Coupled Cluster Framework. *J. Chem. Theory Comput.* 12, 4778-4792 (2016).
35. Garcia-Ratés, M., Becker, U., & Neese, F. Implicit solvation in domain based pair natural orbital coupled cluster (DLPNO-CCSD) theory. *J. Comput. Chem.* 42, 1959-1973 (2021).
36. Altun, A., Neese, F., & Bistoni, G. Local energy decomposition analysis of hydrogen-bonded dimers within a domain-based pair natural orbital coupled cluster study. *Beilstein J. Org. Chem.* 14, 919-929 (2018).
